# The English Sublexical Toolkit: Methods for indexing sound–spelling consistency

**DOI:** 10.3758/s13428-024-02395-3

**Published:** 2024-04-09

**Authors:** Robert W. Wiley, Sartaj Singh, Yusuf Baig, Kristin Key, Jeremy J. Purcell

**Affiliations:** 1 Department of Psychology, University of North Carolina at Greensboro, 296 Eberhart Building, Greensboro, NC 27402 USA; 2 Department of Cognitive Science, Johns Hopkins University, Baltimore, MD USA; 3 Maryland Neuroimaging Center, University of Maryland, College Park, MD USA

**Keywords:** Sublexical processing, Consistency norms, Phoneme-grapheme mapping, Reading, Spelling

## Abstract

This work introduces the English Sublexical Toolkit, a suite of tools that utilizes an experience-dependent learning framework of sublexical knowledge to extract regularities from the English lexicon. The Toolkit quantifies the empirical regularity of sublexical units in both the reading and spelling directions (i.e., grapheme-to-phoneme and phoneme-to-grapheme) and at multiple grain sizes (i.e., phoneme/grapheme and onset/rime unit size). It can extract multiple experience-dependent regularity indices for words or pseudowords, including both frequency indices (e.g., grapheme frequency) and conditional probability indices (e.g., grapheme-to-phoneme probability). These tools provide (1) superior estimates of the regularities that better reflect the complexity of the sublexical system relative to previously published indices and (2) completely novel indices of sublexical units such as phonographeme frequency (i.e., combined units of individual phonemes and graphemes that are independent of processing direction). We demonstrate that measures from the toolkit explain significant amounts of variance in empirical data (naming of real words and lexical decision), and either outperform or are comparable to the best available consistency measures. The flexibility of the toolkit is further demonstrated by its ability to readily index the probability of different pseudowords pronunciations, and we report that the measures account for the majority of variance in these empirically observed probabilities. Overall, this work provides a framework and resources that can be flexibly used to identify optimal corpus-based consistency measures that help explain reading/spelling behaviors for real and pseudowords.

## Introduction

A fundamental component of the cognitive processes of reading and spelling relates to how the mental representation of phonological word forms are associated with their orthographic counterparts. This work introduces the English Sublexical Toolkit, a suite of tools designed to support empirical investigations of the multimodal (e.g., orthographic and phonological) sublexical structures of the English language. As with previous researchers cited in this work, we simply use a set of corpus statistics reflecting the empirical reality of different sublexical units in the English lexicon. While we are not the first to highlight the importance of experience for developing sublexical knowledge, what is novel about the approach used to develop the toolkit is that it considers the implications of experience-dependence to a broader and deeper extent compared to previous work. In essence, the toolkit does not just provide consistency measures for a finite list of real words; rather, it is a whole framework for deriving measures of sublexical consistency (and frequency) according to different assumptions about the underlying nature of sublexical representations, which can then be validated against empirical data by determining which assumptions most improve explanatory power. For example, considering the implications of experience-dependence more deeply lead us to introduce here the completely novel measure of “phonographeme frequency” (e.g., that 38 words in the corpus have the grapheme [CC] pronounced /k/ and only two have it pronounced /tʃ/), which we report explains significant amounts of variance in reading behaviors, for both real words *and* pseudowords. Altogether, we provide a novel operationalization of experience-dependence in the study of the statistical regularities of the sublexical system, and doing so allows us to consider novel implications of this old idea.

Questions about sublexical processes have most often been addressed in the context of reading, where a number of theoretical models have been proposed in the past decades (for a review, see Rayner & Reichle, 2010) – most prominent among them are two classes of models: localist “dual-route” models and connectionist “triangle” models. In dual-route approaches, such as the influential dual-route cascaded (DRC) model (Coltheart et al., 2001), access to phonological forms from orthographic input can proceed through either of two processes, known as the lexical and sublexical routes. Through the lexical route, also known as the “addressed” route (e.g., Patterson, 1986; Coltheart et al., 1991), whole-word phonological representations are accessed via recognition of whole-word orthographic representations, such as [CAT] to /kæt/ (specific models differ on whether or not they posit mandatory intermediate access through lexical semantics; see, e.g., Rapp et al., 2001). In contrast to the lexical route, the sublexical or “assembled” route allows for a process known as “spelling-to-sound” or “grapheme-phoneme” conversion, wherein access to phonology is achieved not through associating unitary, holistic representations of the whole word but rather through orthographic and phonological units of some grain size *smaller than the whole word*, such as [C] to /k/ followed by [AT] to /æt/.

Juxtaposed to dual-route models, triangle models eschew any distinction between lexical and sublexical processes, instead positing a network of interconnected orthographic, phonological, and semantic units (the three vertices of the triangle). As a consequence, whereas dual-route models represent lexical knowledge (as opposed to sublexical) in discrete units, triangle models represent it as distributed across the connections between the processing units. While these two approaches to understanding reading exhibit fundamental differences, one thing that they have in common is the concept of sublexical representations – orthographic and phonological units smaller than the whole-word – that are used to generate plausible pronunciations for previously unencountered words. Moreover, while these theories were developed in the context of reading, models of spelling function largely by analogy (on the relationship between reading and spelling, see for example Holmes & Carruthers, 1998; Rapp & Lipka, 2011; Shanahan, 2016), and the ability to generate plausible spellings for novel words is also thought to be supported via these sublexical units. However, the exact nature of these sublexical representations is not well understood, even though they have been considered critical for learning to read and spell (e.g., Apel et al., 2019; Gough & Tunmer, 1986), are implicated in deficits such as dyslexia and dysgraphia (e.g., Beeson et al., 2000; Monsell et al., 1992; Rapp et al., 2002), and are key to understanding cross-linguistic differences (e.g., the relative contributions of sublexical and lexical processing vary markedly across languages, e.g., Frost et al., 1987; Ziegler & Goswami, 2005).

Recently, there have been a number of efforts to more thoroughly characterize the inner workings of sublexical processes and their relationship to lexical processes (e.g., Chee et al., 2020; Siegelman et al., 2020; Siew & Vitevitch, 2019). For example, the size of the representational units at work in the sublexical route has been examined from relatively fine-grained, individual grapheme-phoneme mappings (e.g., [C] ➔ /k/) to relatively coarse-grained mappings, such as the rime (e.g., [AT] ➔ /æt/) or oncleus (e.g., [CA] ➔ /kæ/). In English, for example, a wealth of previous research has indicated that the rime contains the most valuable information from an information theory perspective (e.g., Treiman et al, 1995; Siegelman et al., 2020), and consequently consistency at the rime level has been a focus when accounting for behavior in both reading and spelling tasks (e.g., Dich, 2014; Weekes et al., 2006; Burt & Blackwell, 2008). However, it is apparent both theoretically and empirically that the rime is not sufficient to account for the entirety of the sublexical process. For example, Burt & Blackwell (2008) reported instances of participants spelling pseudowords with novel orthographic rimes, despite those pseudowords having extant phonological rhymes (e.g., /soʊb/ spelled SOAB, despite all rhyming words in English being spelled -OBE). Moreover, the mere fact that one can generate a spelling for a previously unattested rhyme in English (such as -/aɪtʃ/ perhaps spelled -ICHE or -YTCH) demonstrates that sublexical processes must operate, at least to some extent, at the lower level of individual phoneme-grapheme mappings.

The directionality of mappings has also been investigated because spelling consistency, p(G|P) (the probability of the graphemes given the phonemes), and reading consistency, p(P|G) (the probability of the phonemes given the graphemes) dissociate in languages like English. For example, given the letter X, the probability that it is pronounced /ks/ is very high (i.e., reading consistency p(P|G) is high). However, the reverse is not true: the spelling consistency p(G|P) for /ks/ spelled X is relatively lower because of the large number of alternative spellings – /ks/ may be spelled KS, or CKS, or CS, etc. (as in TREKS, PACKS, or EPICS). This phenomenon has been most extensively studied in the context of reading, where it is described as “feedforward” consistency from graphemes to phonemes; however, “feedback” spelling consistency (from phonemes to graphemes) has repeatedly been shown to affect behaviors such as naming latency and lexical decision (see e.g., Ziegler et al., 2008).

In order to better understand these various phenomena in written language processing (consistency effects, grain-size, feedback, etc.), researchers need detailed measures of the units of sublexical representation – in particular, their consistency and frequency. While a number of options currently exist for researchers to collect such measures, they are limited in many ways. For example, the available databases are either in the form of lists of real words with consistency measures (computed in different ways, depending on the database) or tables of correspondences (e.g., the consistency of the rhyme /oʊp/ spelled [OPE] versus [OAP]). Here, we present the English Sublexical Toolkit, a set of tools designed to quantify sublexical units in multiple ways, serving both practical and theoretical purposes. The central premise of the Sublexical Toolkit is that sublexical representations are primarily acquired through learning the associations between orthographic and phonological segments in the context of real words, not through an explicit system of “rules” for converting between letters and sounds or a consciously prescribed set of correspondences. Consequently, a full understanding of the mental representations subserving written language processing requires detailed measures that empirically examine the regularities within the lexicon. The current version of these tools provides both consistency and frequency measures for two levels of granularity: low-level mappings between individual phonemes and individual graphemes, henceforth *phonographemes*, and higher-level mappings between onsets (syllable-initial consonants) and *rimes* (vowels plus syllable-final consonants). The consistency measures are available in both the spelling p(G|P) and reading p(P|G) directions in the form of continuous measures, reflecting the probability that a given phoneme will be spelled with a certain grapheme (and vice versa), ranging from near 100% (e.g., /b/ is nearly always spelled [B]) to near 0% (/ɹ/ is rarely spelled [RH]).

There are three particularly novel aspects to this work: First, these measures are accessible in the form of toolkits that allow the user to input grapheme-phoneme/phoneme-grapheme mappings to extract measures for *any* word, including pseudowords and misspellings. To the best of our knowledge, this is the first tool that provides a method for readily computing consistency measures for *any* string of letters and phonemes, not restricted to a finite list of real words. This capability in particular enables new opportunities for assessing behaviors with pseudoword tasks, scoring errors (misspellings/mispronunciations), and generating novel stimuli with desired properties.

Second, some of the measures themselves are entirely novel, a consequence of the experience-dependent, corpus-based framework adopted in the current work. In particular, we present original measures of the *frequencies* of sublexical units, including multi-letter graphemes (e.g., [OUGH]) that are distinct from available unigram, bigram, or trigram frequency measures, as well as “phonographeme” units – the co-occurrences of individual phonemes and individual graphemes (e.g., [CH] pronounced /tʃ/ versus [CH] pronounced /k/). The term “phonographeme” is used throughout this work in a non-directional sense (e.g., it is not juxtaposed with a “graphophoneme”). The concept of a sublexical unit common to both reading and spelling is made clear by examining how consistency and frequency are computed (Eqs. 1–3):

1 $$Reading\; Consistency\; p\left(P|G\right)= \frac{\# of\; words\; with\; grapheme\; X\; mapped\; to\; phoneme\; Y}{\#\boldsymbol{ }{\varvec{o}}{\varvec{f}}\boldsymbol{ }\;{\varvec{w}}{\varvec{o}}{\varvec{r}}{\varvec{d}}{\varvec{s}}\boldsymbol{ }\;{\varvec{w}}{\varvec{i}}{\varvec{t}}{\varvec{h}}\boldsymbol{ }\;{\varvec{g}}{\varvec{r}}{\varvec{a}}{\varvec{p}}{\varvec{h}}{\varvec{e}}{\varvec{m}}{\varvec{e}}\boldsymbol{ }\;{\varvec{X}}}$$

2 $$Spelling\; Consistency\; p\left(G|P\right)= \frac{\#\; of\; words\; with\; grapheme\; X\; mapped\; to\; phoneme\; Y}{\#\boldsymbol{ }{\varvec{o}}{\varvec{f}}\boldsymbol{ }\;{\varvec{w}}{\varvec{o}}{\varvec{r}}{\varvec{d}}{\varvec{s}}\boldsymbol{ }\;{\varvec{w}}{\varvec{i}}{\varvec{t}}{\varvec{h}}\boldsymbol{ }\;{\varvec{p}}{\varvec{h}}{\varvec{o}}{\varvec{n}}{\varvec{e}}{\varvec{m}}{\varvec{e}}\boldsymbol{ }\;{\varvec{Y}}}$$

3 $$Phonographeme\; Frequency= of\; words\; with\; grapheme\; X\; mapped\; to\; phoneme\; Y$$

The first two equations are specific to the reading and spelling processes and yet they share their numerator; it is only the denominator that distinguishes between reading and spelling consistency. This follows from the fact that when one experiences a word with a particular grapheme-phoneme mapping, one is simultaneously experiencing a word with the equivalent phoneme-grapheme mapping: for example, an instance of reading aloud the word “cat” entails both experiencing the grapheme-phoneme mapping [C] ➔ /k/ *and* the phoneme-grapheme mapping /k/ ➔ [C] – in other words, feedback processing ensues feedforward processing, and so the phonographeme frequency of the [C] ➔ /k/ mapping is the same as that of the /k/ ➔ [C] mapping: simply the number of occurrences of [C] ➔ /k/ (which are simultaneously occurrences of /k/ ➔ [C]).

Finally, the tools were made with specific theories of sublexical representations in mind, and as such, we present in detail how they were constructed in terms of those underlying theories. Doing so will better enable us to interpret the empirical data and draw inferences from the relative success or failure of the measures (relative to each other and relative to alternative measures developed by other researchers). Moreover, we are making the tools and codes openly available to researchers so that they can be adapted to test new hypotheses (e.g., alternative rules for syllabic parsing or theories of position coding), and/or extend their capabilities (e.g., to other grain sizes or to other languages).

The remainder of this paper is divided into three sections. The next section (Methods) details the methods used to construct the English Sublexical Toolkit, describing all of the procedures taken and decisions made when computing the various measures of consistency and frequency. The third section (Empirical validations) presents three sets of analyses to validate the toolkit measures (construct validity). It does so by: (1) exploring the similarity of our measures to some of the most commonly used consistency measures previously published in the literature; (2) assessing how well our measures explain naming and lexical decision data from the English Lexicon Project (ELP; Balota et al., 2007); and (3) presenting a novel analysis that accounts for the distribution of different pronunciations in response to a pseudoword reading task. The paper concludes with a discussion (General discussion) highlighting the current capabilities of the toolkit, its limitations, and future directions. A number of appendices are also provided, including vignettes that serve as practical guides for how to use the toolkit.

## Methods

Determining the empirical regularities of the English written lexicon requires a large number of decisions about how to operationalize the measures. One contribution of the current effort is to make those decisions explicit and to ground them in cognitive theory. It is challenging to identify all the decisions that must be made, given that they may be hidden by virtue of being based on implicit assumptions. Nonetheless, this section seeks to provide transparency into how the Toolkit was designed to develop a rigorous, coherent system for quantifying English sublexical regularities. The goal is to articulate the theoretical underpinnings of the toolkit such that they generate specific, testable claims that can provide new insights into the underlying nature of sublexical representations in written language. Both the successes and the failures of the toolkit in explaining empirical phenomena allow for testing the theories that informed how the measures were developed. Moreover, future versions of the toolkit can be created by making alternative decisions based on competing hypotheses, thereby allowing for empirically derived support for one hypothesis relative to another. For example, the consistency measures could be recomputed after parsing words according to an alternative to the maximum onset principle (MOP; Kahn, 2015), and meaningful inferences could be drawn if it were found that the alternative resulted in the toolkit better fitting some type of reading or spelling behavior. This type of future work includes many possibilities that will be returned to in the General discussion, including specific examples of how alternative or extended toolkits might be used. The remainder of this section describes each of the major decisions that were made in developing the toolkit, with specific attention paid to the theoretical implications.

### Overview of the English Sublexical Toolkit

**Fig. 1:**
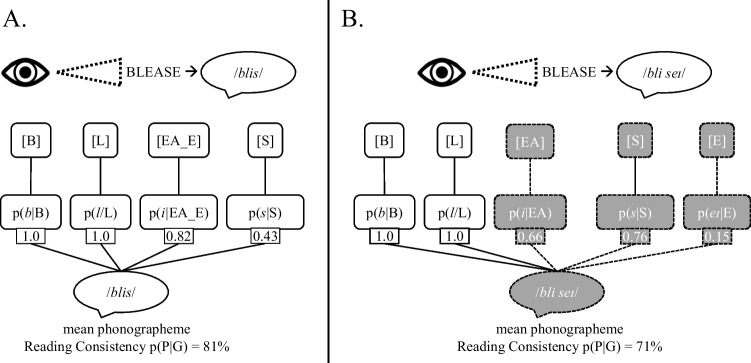
The sublexical system as conceptualized in the English Sublexical Toolkit at the phonographeme level, here visualized during the process of reading aloud the visually presented pseudoword BLEASE. **A**: given a monosyllabic parse with all of the orthographic vowels as a single grapheme (EA_E). **B**: given a disyllabic parse with EA in the first syllable and a *non*-silent E in the second syllable (BLEA-SE). The numeric values reflect the reading consistency for that segmental mapping p(P|G). For example, 82% of words with EA_E in the middle of a syllable are pronounced /*i*/. The *mean* consistency is higher for the monosyllabic reading (81%) compared to the disyllabic (71%), averaging over all segments; this is largely due to the much lower *minimum* consistency (the single least-consistent segment) of the disyllabic reading (just 15% for word-final [E] pronounced /eɪ/). Graphemes are represented in [brackets], phonemes in /*slashes*/. p(P|G) = reading consistency. *Gray fill* indicates alternative parsings/mappings (e.g., EA_E versus EA and non-silent E)

**Fig. 2:**
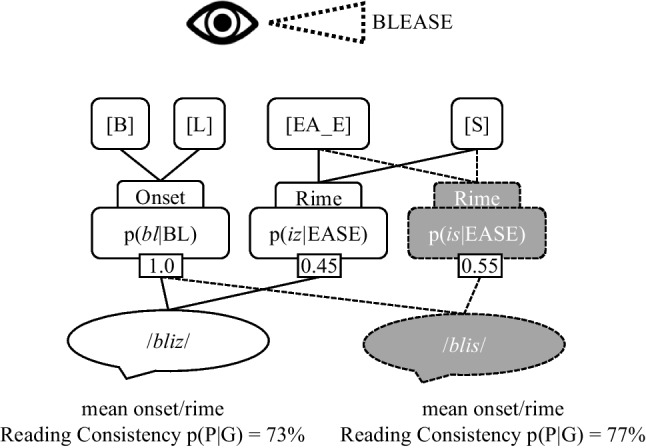
The sublexical system as conceptualized in the English Sublexical Toolkit at the onset/rime level, here visualized during the process of reading aloud the visually presented pseudoword BLEASE, for two alternative phonemic assignments given the same orthographic parsing (/blis/ versus /bliz/). *Gray fill* indicates alternative parsings/mappings (e.g., the rime -EASE read /iz/ versus /is/). Graphemes are represented in [brackets], phonemes in /*slashes*/. p(P|G) = reading consistency

An overview of the sublexical processes as conceptualized in the Sublexical Toolkit at the phonographeme level is presented in Fig. 1, with an example of reading aloud the visually presented pseudoword BLEASE. The toolkit is available freely from the Open Science Foundation (OSF) at https://osf.io/e95qw/?view_only=167fb28c4842491a885b91435c57b2f0. An overview of the onset/rime level is presented in Fig. 2. The distinction between the two regards the grain size of the representations under examination: at the lowest level, phonographemes (individual phoneme-grapheme mappings) are measured, and at the higher level the units are onsets (syllable-initial consonants, including clusters like CHR or PHL) and rimes (vowels plus following consonants, i.e., the syllabic nucleus and coda). Throughout the manuscript we focus on the reading process, but it should be noted that the toolkit provides both reading consistency p(P|G) and spelling consistency p(G|P) measures. It will be explicitly noted whenever the two directions of processing are importantly distinguished in terms of how the toolkit functions.

In general, computational models of English reading/spelling must address both parsing of inputs and mapping to outputs (e.g., Gubian et al., 2022; Mousikou et al., 2017; Perry et al., 2007; Pritchard et al., 2012). Models such as the connectionist dual -route model of Perry and colleagues (CDP++, Perry et al., 2010) and the DRC model of Rastle and Coltheart (Rastle & Coltheart, 2000) can generate pronunciations for pseudowords given letters as input by using heuristics to determine how the letters should be grouped into graphemes and what phonemes they should be paired with. The core work of the toolkit presented here is focused on the mappings of phonemes and graphemes but does not have a deterministic heuristic or algorithm that dictates how letters “should” be combined into graphemes or which phonemes should be used. Rather, given a *predefined* spelling-sound mapping, the toolkit extracts how consistent that choice is with the English lexicon (currently based on a sample of ≈14,000 words). This means that the toolkit’s user must provide both the graphemes and the phonemes, whereas the computational models of reading require only input graphemes to then generate a phonological output.

For example, presented with the pseudoword ADANE, most English speakers will pronounce it /æ 'deɪn/ (Mousikou et al, 2017), indicating that the word was parsed as two syllables, A-DANE, with the final -E in the second syllable grouped with the preceding A as an A-silent-E; we refer to such mappings with an underscore, as in A_E. Given the input ADANE, the DRC model reads the string as /ə 'deɪn/ (Mousikou et al., 2017), differing in the pronunciation of the first letter but otherwise agreeing with the most common human response and the mapping of A_E ➔ /eɪ/. The toolkit does not strictly generate a single pronunciation, but rather can be used to measure the consistency of any given response. For the pseudoword ADANE, for example, at the onset/rime level the toolkit indicates that the most popular human pronunciation is indeed more consistent than is the DRC pronunciation: the mean p(P|G) at the onset/rime level of granularity is 77% for the human response, versus 73% for the DRC response. That is, given the orthographic parse A-DANE, the pronunciation /æ 'deɪn/ is more plausible than /ə 'deɪn/. The current version of the toolkit cannot be used to determine the probability of the parse itself, such as whether a disyllabic parsing A-DANE is more or less likely than a trisyllabic parsing A-DA-NE. However, it can say how consistent A-DA-NE pronounced /'æ də ˌneɪ/ is compared to another pronunciation with the same parsing such as /ə 'dɑ ni/.

The toolkit operates under the self-evident assertion that the knowledge of sound–spelling mappings is grounded in experiential learning. This implies that the relative strength of these connections depends on the amount of experience individuals have with these representations, and this strength in turn can be estimated from the regularity with which sublexical mappings of various grain sizes occur in the English lexicon. This assertion is supported by the preponderance of evidence that pseudoword responses vary greatly both across and within individuals (e.g., Coltheart & Ulicheva, 2018; Ulicheva et al., 2021). Importantly, this variability is much greater than would be expected if the sublexical system operated in a rule-based way that only considers mappings with a high probability, or that requires adherence to larger units such as rimes that are extant in the lexicon. There is clear evidence that individuals generate pseudoword spellings that result in previously unattested rimes – for example, the pseudoword /snoʊb/ has been spelled SNOAB (Burt & Blackwell, 2008), despite the fact that *all* rhyming words in English are spelled -OBE (thus one expects the spelling SNOBE). We take such evidence as indication that phonographemes are productive units of representation, although their relative importance compared to higher level units like rimes remain an open question.^1^

Altogether, the toolkit quantifies the probability of spelling-sound mappings, both as *consistency* (the probability of the phoneme given the grapheme, or vice versa) and as *frequency* (the frequency with which that phonographeme is encountered in the lexicon). For example, in Fig. 1, the p(P|G) *reading consistency* value 0.82 refers to the proportion of words with EA_E in the syllable medial position that are pronounced /i/. This was computed by dividing the number of words with *that* pronunciation (e.g., PLEASE, RELEASE) by the total number of words with that *or any other* pronunciation (e.g., HEARSE, MILEAGE). The toolkit also offers *frequency* measures that are equivalent simply to the numerator of the consistency measure (i.e., the number of words with that mapping, without dividing by the total number of words with that or any alternative pronunciation). Figure 1 shows that the measures are position-specific: for the pronunciation /blis/ the word-final S ➔ /s/ consistency is 0.43, whereas for the pronunciation /bli `seɪ/ the syllable-initial S ➔ /s/ consistency is 0.76, reflecting the fact that words ending in S are somewhat more often pronounced /z/, whereas syllables beginning S are most often pronounced /s/.

Just as the consistency of a mapping varies depending on its position in the word, consistency at the level of onsets and rimes can differ from the phonographeme level due to the idiosyncrasies of English spelling. One consequence is that the most probable reading at one level may differ from that at another level. For example, the pseudoword BLEASE (Fig. 1) is most probably pronounced /bliz/ when averaging over all phonographeme units, which is more probable than the pronunciation /blis/ because word-final S is more often pronounced /z/ than /s/ (57% versus 43%). However, the situation reverses at the level of the onset and rime, as the rime -EASE is more often pronounced -/is/ as in LEASE than -/iz/ as in PLEASE (55 vs. 45%). In this example, empirical behavioral data (Pritchard et al., 2012) indicates that English speakers have nearly a 2:1 preference for /bliz/ over /blis/, a result more in agreement with the phonographeme than the rime consistency. However, it is generally an open question as to whether one or both of these levels determines behavior, and the extent to which it depends on individual differences or other properties of the stimuli (e.g., orthographic neighbors). Regardless, this is one example of how the toolkit can be used to probe such questions.

The remainder of this section describes how orthographic and phonological representations are conceptualized in the toolkit, and the details of a number of specific decisions and assumptions necessary to operationalize this schema into a working tool.

### Corpus

The most basic assumption of the work here is that sublexical knowledge (of sound–spelling mappings) derives from experience with lexical items – consequently, measures of consistency and frequency fundamentally depend upon which words are included in the corpus.

The initial version of the toolkit (version 1.0) is based on approximately 10,000 words, which has been expanded to over 13,000 in the latest update (version 1.1). The initial corpus was formed from three components: all of the monosyllabic words previously coded for Friends/Enemies measures (F/E; Plaut et al., 1996), in order to compare the English Sublexical Toolkit’s measures to the F/E measures; a list of several hundred words that were administered by the authors in other studies of spelling, selected for reasons unrelated to the Sublexical Toolkit; and the rest were the most frequent English words according to the SUBTLEX-US database (Brysbaert & New, 2009). The version 1.1 corpus adds an additional 2688 (the next most-frequent words per the SUBTLEX-US database). The automated R-code based on the version 1.0 corpus successfully parses 99.0% of the words added in version 1.1, which is an indication that even the smaller corpus generalizes to most unseen words. Further details regarding diagnostics of the sufficiency of the corpus are presented in Appendix 3.

### Parsing

Perhaps the most fundamental issue to resolve when measuring sublexical properties is how to segment the string into constituents (i.e., how does the lexical item decompose into sublexical units?). There are three aspects to this issue: parsing the phonological word form into sublexical units, parsing the orthographic word form into sublexical units, and encoding the position of these units within their respective strings. For phonological parsing, we adopted the Maximum Onset Principle (MOP, Kahn, 2015; see also Chee et al., 2020), which has the advantage of being well grounded in theories of phonology as well as being readily operationalized for the purposes of sublexical spelling-sound mappings. This principle determines the location of syllabic boundaries by placing consonants in the onset position (i.e., as the start of a new syllable) unless doing so would lead to a phonotactically illegal utterance in English. That phonotactic legality is determined by the sonority hierarchy, which allows consonant clusters so long as they are patterned as follows: the initial phoneme is /s/, which may be followed by a stop such as /p/ or /n/, which may be followed by a liquid /l/ or /ɹ/, or by a glide /j/ or /w/. For example, LOBSTER is parsed as LOB-STER and not LO-BSTER, because the cluster BST would violate the sonority hierarchy (/b/ cannot precede /s/), nor LOBS-TER, because the S should begin the second syllable (/s/ *can* precede /t/). Application of the MOP results in an internally consistent framework for parsing a string of phonemes (a lexical item) into syllables, and those syllables are defined by consonants (optionally) in the onset, a vowel (mandatorily) in the nucleus, and consonants (optionally) in the coda. This also then determines the onsets and rimes, the latter being simply the concatenation of vowel in the nucleus and any following consonants in the coda (see Fig. 2). It is also worth highlighting that the earlier work of Hanna et al. (1966) and the updates to that work by Fry (2004) and Berndt et al. (1987), suffer from inconsistencies in how word forms were parsed into syllables – and these inconsistencies necessarily had an impact on the measures of spelling-sound consistency. For example, SATYR was parsed as SAT-YR, which not only fails to follow the MOP but also contradicts the parsing of MARTYR (parsed as MAR-TYR).

### Position coding

While the MOP provides a clear guide to parsing the words’ phonology, it does not address how to determine equivalency of sublexical positions. The question of letter position encoding has been highly researched yet remains unresolved (see, e.g., Baciero et al., 2022; Gomez et al., 2008; Grainger, 2018; Grainger & Van Heuven, 2004). To the best of our knowledge, no parallel research has investigated the encoding of position in the context of cross-modal mappings, i.e., between the position of graphemes and the position of phonemes (or higher-level units). In the seminal work of Hanna and colleagues (1966), sublexical mappings were considered in three positions: syllable-initial, medial, or final. In the recent work of Chee and colleagues (2020), positions were measured in serial order from the first syllable (e.g., onset of the first syllable, onset of the second syllable, the third, etc.). These approaches have serious consequences when computing spelling-sound consistency. For example, the serial order position schema implies that the phonographeme /tʃ/ ➔ CH in the word CHAIR is independent of the one in MA-CHETE, and both of those are independent of the one in O-VER-CHARGE (as they are the onset of the first, second, and third syllables, respectively). In the syllable-initial/medial/final scheme, on the other hand, all of those /tʃ/ ➔ CH mappings are treated as being in the *same* position (syllable-initial).

When mapping phonographemes, the English Sublexical Toolkit does not use the serial position schema, as there is currently no particular evidence to support that sound–spelling mappings are represented in this way. Indeed, there are reasons to question the plausibility of a serial position schema, which would imply (among other things) that learning the /tʃ/ in WHICH is spelled CH does nothing to inform one about the potential spelling of the /tʃ/ in OSTRICH (simply because the former is the coda of the first syllable whereas the latter is the coda of the second). Instead, the Sublexical Toolkit adopts and extends the scheme of Hanna and colleagues (1966) from three to five categories: word-initial, syllable-initial, syllable-medial, syllable-final, and word-final. The additional distinction is whether the syllable is the first or last in the word; this was done based on the empirical observation that certain mappings *never* occur word-initial/final but do occur syllable-initial/final if the syllable is internal. This both-ends scheme has found support both in studies of letter position in reading (e.g., Fischer-Baum et al., 2011) and spelling (e.g., Fischer-Baum et al., 2010) and verbal working memory (e.g., Henson, 1999). For example, parsing the word HAPPY by the MOP results in syllabification as HA-PPY, and as such the /p/ maps to PP. Per the three-position syllabic schema of Hanna and colleagues, /p/ ➔ PP is legal for initial positions – however, it is immediately apparent this is not true for the start of a word. Therefore, in the Sublexical Toolkit the mapping /p/ ➔ PP is possible for syllable-initial, but not word-initial, mappings.

An analogous distinction is made at the onset/rime level: onsets are either word-initial or syllable-initial (onset of a second or later syllable), and rimes are either word-final or syllable-final (rime of a penultimate or earlier syllable). This effectively addresses the issue of how to handle rhymes in multisyllabic words – monosyllabic words are all treated as having only a word-final rime, whereas multisyllabic ones are composed of one or more syllable-final rimes and a single word-final rime.

### Pronunciation

While a word’s correct spelling is not subject to debate (alternative spellings or American/British differences notwithstanding), the same is not true of pronunciation. There are multiple sources of variability in speakers’ pronunciation, including regional differences, social class, gender, age, and education (Rickford, 1996). Any measure of English spelling-sound consistency must grapple with the challenge presented by the fact that there is no monolithic, universal English language. For example, the well-documented cot-caught merger (e.g., Labov et al., 2006) entails that speakers who pronounce such words identically must have less consistent sound–spelling mappings compared to those who do not have the merger, as the merger of the two vowels /ɑ/ and /ɔ/ is not reflected in orthography^2^. Indeed, an intriguing direction for future research is to determine the extent to which individual differences in reading/spelling are attributable to idiosyncratic pronunciation (i.e., person-specific “accents”), which may affect the degree to which English sound–spelling mappings are perceived as (in)consistent. Individuals whose internal phonological representations are more consistently reflected in standardized spelling may be at an advantage for learning to read and spell, relative to those whose dialects are more opaquely related to spelling.

In any case, across-speaker variability in pronunciation limits the ability of any consistency measure to account for behavioral data to the extent that the population sampled will vary in how well it matches with the “canonical” pronunciations used to inform the consistency measure. Indeed, this is a considerable limitation of the database of Hanna and colleagues (1966), as it is apparent that not all of the phonological representations in their corpus reflect contemporary standard American English. For example, the AY in *yesterday* was mapped to the same vowel as the Y in *baby* and the UI in *guitar* – but contemporary standard American pronunciation maps the AY ➔ /eɪ/, the Y ➔ /i/, and the UI ➔ /ɪ/. To address this issue, the work at hand adopted two primary sources for determining the underlying phonology in the corpus data used to construct the consistency measures. Specifically, the Carnegie Mellon Pronouncing Dictionary (Weider, 2005) was used as the primary determinant of lexical phonology, and in instances where there were multiple pronunciations, all were included in the corpus, regardless of whether the alternatives were homographs (as in BASS /beɪs/ versus /bæs/) or regionalisms (as in PECAN /pi kɑn/ versus /pɪ kæn/). In instances where there was an apparent error in the dictionary or a missing entry, the Cambridge English pronouncing dictionary (American accent) was consulted (Jones, 2011).

### Morphology

While we acknowledge the evidence that sublexical and lexical processes are interactive in nature, the measures presented here are designed to reflect sublexical processes independent of lexical influences. In particular, the consistency measures do not consider morphological representations of any form, inflectional or otherwise. This is necessary when applying the MOP to parse the phonology, because the resulting syllabic boundaries will routinely be at odds with morphologically defined boundaries (e.g., the MOP parses EATER as EA-TER, whereas morphologically the boundary is EAT-ER).

It is certainly true that lexical knowledge, such as knowing a word’s part of speech, will influence performance on tasks that require sound–spelling mappings, but by definition it will not inform the sublexical *processes* that the toolkits are intended to reflect. For example, the heard-pseudoword /klaɪd/ presented as a past-tense verb (“She /klaɪd/ the toy on the ground.”) is more likely to be spelled ending in -ED, such as CLIED, than when presented as a noun (“She bought two pounds of /klaɪd/), such as CLIDE. We do not consider it a limitation of the toolkit that it does not integrate such information, because it is designed to reflect stages of processing that occur prior to, or perhaps are encapsulated from, lexical influences. Future work will determine how morphological, semantic, and syntactic representations impinge upon reading and spelling processes even during pseudoword tasks, but this is beyond the scope of the current toolkit.

### Graphemes

One fundamental assumption we make is that the sublexical system requires a one-to-one mapping of graphemes to phonemes. It is obvious that English does not have a one-to-one mapping of *letters* to phonemes (e.g., consider the homophones NIGHT and KNIGHT), which may be why there is the common conception of “silent letters” and many studies on how silent letters are “read” (e.g., Ehri & Wilce, 1982; Gingras & Sénéchal, 2019; Perry et al., 2014). However, we conceptualize the sublexical system as requiring that every *grapheme* be mapped to a pronunciation, and thus in this sense there are no “silent letters”, only graphemes whose pronunciations differ from those of their constituent letters. For example, in the word WEIGH there are just two phonemes, /w/ and /eɪ/ – in the Sublexical Toolkit, the mappings are /w/ ➔ W and /eɪ/ ➔ EIGH. One alternative framework might map the GH as “silent”, but such a framework would face the serious challenge of how to determine which letters are pronounced and which are “silent”.

In the recent work of Chee et al. (2020), a relatively small inventory of consonant graphemes was used, with all other letters assigned to the vowel graphemes. For example, the only grapheme corresponding to /m/ is listed as M (Chee et al., 2020, Table 11) – it is unclear how this allows for accounting for words like COMMA, LAMB, or DAMN. In the earlier works of Fry (2004), Berndt et al. (1987), and Hanna et al. (1966), a larger inventory of graphemes was used, presumably on the original basis of the procedure of Hanna and colleagues that focused on spelling (i.e., given their corpus of ≈17,000 words, they listed all graphemes needed to spell the phonemes in that corpus). For example, /m/ corresponds to potentially M, MM, MN, and LM (Fry, 2004). The Sublexical Toolkit began with the graphemic inventory originated by Hanna and colleagues (1966) but made parsimonious adjustments over the course of incorporating words into the corpus. *Specifically, letters were assigned to graphemes such that the final inventory had as few unique graphemes as possible*. For example, ROGUE could potentially be parsed in three ways:

1. With /g/ ➔ GUE
2. With /g/ ➔ G, and so consequently the vowel /oʊ/ ➔ O_UE
3. With /g/ ➔ GU and /oʊ/ ➔ O_E

The first option was employed by Hanna and colleagues and was restricted to syllable-final positions as in RO**GUE** and MOR**GUE**. The second option is appealing because it allows the /g/ to have the most common spelling, G, but it is unappealing because it requires positing a grapheme made of noncontiguous letters (/oʊ/ ➔ O_UE). That problem would be exacerbated by encountering other vowel mappings in a similar context; for example, LEAGUE would require an EA_UE grapheme. Instead, the third option was adopted for the Sublexical Toolkit based on the principle of parsimony (Epstein, 1984), because it minimizes the total number of graphemes required. It does so because both the GU and O_E graphemes are necessary for *other* words – the GU as in GUESS (not included in the original Hanna et al., 1966 nor the Berndt et al., 1987, but adopted by Fry, 2004) and the O_E as in GONE.

In other words, the third option allows for the words ROGUE, GUESS, and GONE to be represented with six graphemes total (R, O_E, GU, SS, G, and N), whereas the first option would require eight (R, O, GUE, G, UE, SS, O_E, and N) and the second option would require at least seven (R, O_UE, G, UE, SS, O_E, and N). This parsimonious approach was taken throughout the process of building the toolkit, keeping the number of graphemes to a minimum while also providing a principled heuristic for whether or not to adopt new graphemes. It is also worth noting that this principle did not necessarily result in grouping orthographic consonants and vowels together to form graphemes as with GU. For example, while QU is sometimes mapped to /k/, as in QUICHE, in other words the Q is mapped separately from the U, as in QUICK, where the U maps to /w/. That did not require positing an additional phonographeme, as the mapping of U to /w/ is not limited to the QU bigram, but also occurs in words like CUISINE, DISTINGUISH, and PERSUADE. All of the graphemes identified for the Sublexical Toolkit that occur at least 1% of the time and in at least two different word forms are presented in Appendix 1 (for vowels) and Appendix 2 (for consonants), as a series of phoneme-grapheme correspondences (e.g., /f/ spelled F, FF, PH, or GH); all graphemes including those occurring very infrequently are accessible through the English Sublexical Toolkit itself available on OSF (https://osf.io/e95qw/?view_only=167fb28c4842491a885b91435c57b2f0).

### Final E’s

One hallmark of English spelling is the prevalence of the “silent E”, referring to when the letter E appears after a consonant but does not receive its own pronunciation. It has long been noted that this E tends to have an effect on the quality of the vowel, typically “lengthening” (see, e.g., Joshi et al., 2008) – hence the distinction between BID /bɪd/ and BIDE /baɪd/. We prefer the term “final E” instead, to highlight the fact that these letter E’s do in fact convey information about pronunciation, and moreover, some are not silent in any sense but instead reflect a non-linear mapping between letters and sounds. As such, there are two categories of final E: those traditionally called “silent E” and those we call “non-linear E”. Instances of the first category are always referred to using underscores, as in the A_E of BAKE or O_E of POSE – these *silent E’s* modify the quality of a preceding vowel. Unlike the silent E’s, *non-linear E’s* do not modify a preceding vowel, but rather represent a vowel themselves, specifically either schwa /ə/ or /ʌ/. This second category of final E is very clearly seen when comparing words such as MUSCLE and MUSSEL. While they are pronounced identically, the /əl/ at the end of the words maps directly (linearly) onto the EL in MUSSEL, whereas the schwa /ə/ in MUSCLE maps onto the final, non-linear E as though the order of the phonemes were reversed (the phonological order is vowel + consonant, but the orthographic order is consonant + vowel). In some works, these are treated as syllabic consonants (including that of Hanna et al., 1966). Within the corpus used to build the toolkit, in addition to the common final -LE ending (TABLE, MAPLE, etc.) we also encountered less frequent but analogous instances of -RE (e.g., THEATRE). We further added to this category the idiosyncratic ONE and ONCE, including words in which they are affixed (e.g., SOMEONE). Consistent with the idea that no letter is truly silent, these E’s were treated as non-linear mappings of /ʌ/. For example, the phonology of ONE, /wʌn/, is mapped as /w/ ➔ O, /ʌ/ ➔ (non-linear) E, /n/ ➔ N.

### The letter X

The letter X is unique in that it is the only instance (in English) of a single grapheme used to represent a consonant cluster, typically /ks/ or /gz/. A consequence of employing the MOP is that X is “divided” across syllables when appearing in multisyllabic words. For example, TAXI ➔ /'tæk si/ implies the X is represented both as the coda of the first syllable (/k/) and the onset of the second syllable (/s/). We adopt the same accommodation to the MOP as Chee et al. (2020) by including the letter X with the earlier syllable, effectively parsing the word as TAX-I. An alternative would be TA-XI, which in fact may be predicted by alternative, orthographic-based, parsing rules – but it would suggest a phonotactically illegal English syllable, /ksi/.

We note that others, including the seminal work of Hanna and colleagues (1966), have at times mapped other graphemes onto phonological units consisting of more than one phoneme, most conspicuously /kw/ ➔ QU (as in QUICK or QUIET). This was never done in the English Sublexical Toolkit, as the phonographeme level of representation is defined to be the smallest plausible mapping, i.e., individual phonemes to individual graphemes – as such, the letter X is the only exception. For example, the /kw/ ➔ QU of Hanna et al. (1966) was decomposed into /k/ ➔ Q and /w/ ➔ U. This decision was also compatible with the criteria for deciding on the graphemic inventory (see “Grapheme inventory and grapheme parsing”), as some words with QU cannot be mapped to /kw/ (as in QUICHE), and some words require /w/ ➔ U even in the absence of Q (as in CUISINE).

## Measures available in the English Sublexical Toolkit

The methods described above were used to construct a system for extracting a number of sublexical regularities from the lexicon along phonological and orthographic dimensions. These methods were applied to a corpus of over 13,000 English words to compute their sublexical sound–spelling consistency and frequency measures. For an analysis of the sufficiency of the corpus size, see Appendix 3. Critically, these words also formed the basis for measuring the consistency/frequency of any hypothetical string of letters mapped to any potential string of phonemes (or vice versa). For example, the rime -ASTE was found to be pronounced as -/eɪst/ in 88% of words in the corpus (e.g., WASTE, TASTE) and as -/æst/ in 12% (e.g., CASTE). The Sublexical Toolkit can be used not only to look up the values for words included in the corpus, such as WASTE and CASTE, but also to compute the values for words not included in the corpus (regardless of their lexical status). This includes pseudowords such as DASTE, which would be most consistent if pronounced as /deɪst/ rather than as /dæst/ (but either pronunciation would be more consistent than, e.g., /dɪst/ or /dust/).

In total, there are ten measures offered by the toolkit: p(P|G) reading consistency, p(G|P) spelling consistency, phoneme frequency, grapheme frequency, and phonographeme frequency, each at the phonographeme and the onset/rime level. They are available both segmentally (e.g., the consistency of the word-final CH ➔ /k/ mapping in STOMACH) and as summary statistics over the whole word (e.g., the mean consistency of STOMACH averaging across S ➔ /s/, T➔ /t/, O ➔ /ʌ/, etc., or the minimally or maximally consistent segment across the word). Currently, all of the measures are weighted by type, not token (e.g., there are two electronic formats of the toolkit, Excel worksheets and R code, which provide the same information but differ somewhat in their ease of use. In general, the R code Sublexical Toolkit is very fast both at processing words and searching for words or segments with desired properties, whereas the Excel worksheets better support detailed inspection of single words.

There are two particularly novel contributions of these tools. First, they can be used to compute the measures for any desired string, because there is no limitation to the corpus of ≈ 14,000 word; rather, those words form the basis on which any strings can be judged as consistent. This is similar to the correspondence tables of Hanna et al. (1966) and the updated versions from Fry (2014) and Berndt et al. (1987), except those have not been converted into digital tools. Moreover, those resources suffer from a number of errors and internal inconsistencies originating in the 1966 work of Hanna and colleagues, as described throughout this Methods section. Second, the methods used to develop the measures are themselves novel – in particular, the frequency measures are unlike any previously published in the literature. While orthotactic unigram, bigram, and trigram frequency measures are available elsewhere (as are analogous phonotactic measures), no database has quantified the frequency of *graphemes*, which are conceptually distinct from those orthotactic units. The *phonographeme frequency* is also an entirely novel measure, as is the concept of non-directional sublexical units. Finally, it is also worth noting that the *phoneme frequency* measure is novel relative to uniphone frequency measures available from phonotactic databases (see Vitevitch & Luce, 2004), because here the phonemes are coded according to the five-position schema (word-initial/final, syllable-initial/medial/final), which has not previously been used to investigate the effects of phoneme frequency.

We have included vignettes and video guides with further details and practical explanations of how to use the various components of the toolkit (both Excel-based and R-based) on OSF at: https://osf.io/e95qw/?view_only=167fb28c4842491a885b91435c57b2f0.

### Empirical validations

The following sections present a series of empirical validations of the toolkit measures: (1) a comparison of the toolkit’s measures with two other sets of measures available in the literature; (2) a series of stepwise regression analyses of English Lexicon Project data (ELP; Balota et al., 2007) to explore the contribution of the toolkit measures to explaining variance in reading behaviors after controlling for surface and lexical variables; and (3) a novel analysis of the pseudoword reading data of Pritchard et al. (2012) to assess the toolkit measures’ ability to account for the within-item variability in pseudoword pronunciations. An internal validation of the Sublexical Toolkit^3^, specifically the sufficiency of the corpus size to establish reliable measures, is also presented in Appendix 3.

## Comparisons with other consistency measures

Previous efforts to develop English sound–spelling consistency measures have varied considerably in their methods. Nonetheless, given that they have had some success in accounting for behavioral data with reaction time and accuracy in lexical decision and oral reading of real words, significant positive correlations are expected between the various measures. Here, we examine the correlations between the set of toolkit measures with two other sets: the Onset and Rime consistency norms from Chee et al. (2020) and the Friends/Enemies (F/E) consistency measures from Plaut et al. (1996). These correlations are useful for understanding the extent to which different methodological choices impact the resulting consistency measures (e.g., how to account for syllabic position, how to parse the graphemes, etc.). In addition, they give indications as to how much consistency measures vary as a consequence of different grain sizes and different directions, p(P|G) and p(G|P).

### Materials

We first identified words that are coded in our database as well as those available in Chee et al. (2020) and F/E measures of Plaut et al. (1996). Of the words in the toolkit database, 9164 were also coded for rime consistency by Chee et al. (2020), and 9016 for onset consistency.^4^ We specifically used the “composite” measures from Chee et al., which are the mean values across all syllables in the word, and so the toolkit measures used for this purpose were also the mean values. For the F/E measure, which by definition corresponds just to the rime, 2861 words were coded in our database – this number is smaller because the F/E measure is computed only for monosyllabic words, whereas the other measures included in this analysis are for both monosyllabic and multisyllabic words.

### Analyses

The Pearson correlation between each set of measures was computed, without correction for multiple comparisons. As reported in Table 1, the vast majority of the correlations that are significant have *p* values < 0.001, and so would survive even stringent corrections such as Bonferroni.

**Table 1:**
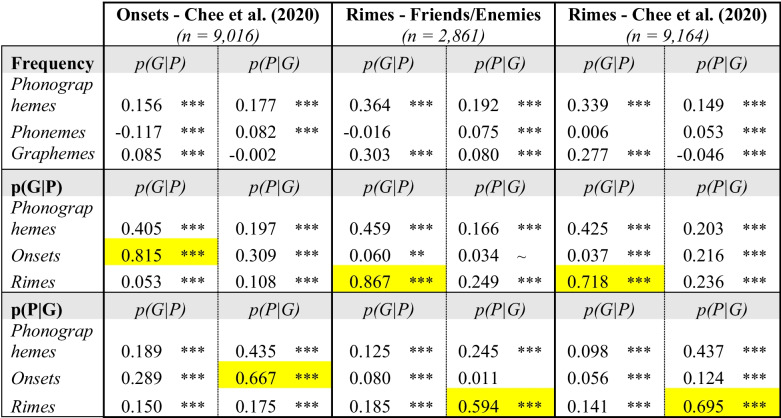
Frequency = toolkit (log) frequency measures. p(G|P) = spelling consistency. p(G|P) = reading consistency. *Highlighted cells* indicate a conceptual match between measures, where the highest correlations are predicted (e.g., toolkit p(G|P) onset with Chee et al. (2020)’s p(G|P) onset consistency. *** *p* < 0.001, ** *p* < 0.01, ~ *p* < 0.10

### Results

As shown in Table 1, most pairwise correlations between measures are significant and positive. Only five correlations are non-significant, three of those being with the Sublexical Toolkit phoneme and grapheme frequency measures – this is not surprising, as there is no particular reason that phoneme or grapheme frequency should be correlated with consistency measures at the level of onsets or rimes. The phonographeme frequency measure is more highly correlated with the consistency measures than the phoneme or grapheme frequency measures, however. The implication is that more consistent spellings are associated with more frequent phonographeme units, which is nearly a tautology (although frequency and consistency are not the same, it stands to reason that more consistent mappings must generally be more frequent in the lexicon).

The cells highlighted in yellow in Table 1 draw attention to the correlations that logically should be the highest, as they represent a conceptual match between the measures: they are the correlations between measures matching in *both* direction *and* grain size. Indeed, these six correlations are the highest overall, ranging from 0.594 (for p(P|G) rime consistency with the F/E measure) to 0.867 (for p(G|P) rime consistency with the F/E measure). The other pattern of note is that the toolkit p(G|P) measures are generally more similar to the F/E p(G|P) measures, whereas the toolkit p(P|G) measures are generally more similar to the Chee et al. p(P|G) measures.

### Summary

Generally, the correlations between conceptually similar methods are high, although in some specific instances perhaps not as high as one might expect. It is worthwhile reviewing the primary differences between the approaches that might account for the discrepancies. The F/E measure is based only on monosyllabic words, which necessarily limits how much it might correspond to any approach that also accounts for multisyllabic words. Moreover, the way in which multisyllabic words influence the consistency measures is different between the approach taken here and that of Chee et al. (2020), due to the way in which position is coded. The approach of Chee and colleagues uses a serial position coding scheme and consequently, although both monosyllabic and multisyllabic words contribute jointly to computing consistency, it is only the *first* syllable of multisyllabic words that have an impact on the consistency values for monosyllabic words. This is unlike the toolkit approach, which uses a both-ends position coding scheme, and consequently it is both the first and the last syllable of multisyllabic words that have an impact on the consistency values for monosyllabic words. As a concrete example, this means that the rhyme -/ɪn/ as in KIN is treated as the same as that in KIN-DRED per the Chee et al. approach (both are first syllable rimes), whereas for the toolkits’ approach it is treated the same as that in NAP-KIN (both are word-final rimes). Which of these approaches is closest to psychological reality remains an open question.

## Regression of ELP data

As a further validation of the toolkits, we conducted a series of stepwise multiple regression analyses using data from the English Lexicon Project (ELP; Balota et al., 2007). The goal of these analyses is both to confirm that the toolkit measures contribute significant unique variance in explaining behavioral data from real word reading tasks (naming and lexical decision), and to demonstrate a theoretical contribution of the various measures. Specifically, the stepwise procedure was used to ascertain the extent to which the theoretical order of precedence of the measures is reflected empirically in their relative importance. In addition, these analyses first control for a number of surface and lexical variables, which is a conservative approach to assessing the importance of the consistency measures.

To complement the stepwise regression, we also present the results of elastic net regression to uncover the relative importance of all the variables: surface, lexical, and sublexical, both the toolkit measures and those from Chee et al., 2020 (the F/E measures were not included as they are available only for monosyllabic words). Elastic net regression can be used to determine an optimized subset of predictors from a larger pool, without any a priori decisions about the order in which variables should be tested, unlike stepwise regression. It also has advantages over other forms of regression in terms of dealing with collinearity, which is important here due to correlations between the various predictors (Tomaschek et al., 2018). Here we specifically use elastic net regression with repeated cross-validation, splitting the data into training and testing sets in order to report a measure of the relative importance when maximally accounting for the behavioral data from the ELP.

### Materials

In total, 9164 words were both available in the ELP data set (Balota et al., 2007) and the toolkit corpus. In the instance of homographs, the pronunciation with the higher consistency was selected for the toolkit measures. Both the Naming and Lexical Decision data were extracted from the ELP, and both reaction time (RT) and accuracy were modeled, resulting in a total of four separate stepwise regressions. In addition to the toolkit measures, the following surface and lexical variables were also retrieved from the ELP database (or elsewhere, as noted in the following): Length (in letters), Ortho_N (number of orthographic neighbors), Phono_N (number of phonological neighbors), Freq_N (mean frequency of orthographic neighbors), Freq_Phono_N (mean frequency of phonological neighbors), OLD (mean Levenshtein distance to the 20 closest orthographic neighbors), OLDF (sum frequency of those neighbors), PLD (mean Levenshtein distance to the 20 closest phonological neighbors), PLDF (sum frequency of those neighbors), NSyll (number of syllables), NMorph (number of morphemes), and LgSUBTLWF (log word frequency, SubtlexUS database; Brysbaert & New, 2009). For the elastic net regression only, all of the consistency measures from Chee et al. (2020) were also included (consistency of the Onset, Nucleus, Coda, Oncleus [onset+nucleus], and Rime, in both the reading and spelling directions). For all of the sublexical measures the mean value, across all segments in the word, was entered, rather than the minimum (or the sum, which is confounded with word length).

### Stepwise regression analyses

Multiple linear regression models were computed in a stepwise fashion, similar to the approach of Chee et al. (2020), in six steps. At each step past the first, sublexical variables were entered both as main effects and as interactions with word frequency (LgSUBTLWF), in consideration of the well-established interaction between lexical frequency and sublexical measures like consistency (e.g., Andrews, 1982; Cortese & Simpson, 2000). In addition, multicollinearity was assessed at each step by the use of variance inflation factors (VIF), computed with the R package *car* (version 3.1-0; Fox and Weisberg, 2019). All of the sublexical variables had VIF scores < 10 when entered at their respective steps in the model^5^.

- *Step one:* The base model included all surface and lexical variables listed above in Materials plus two toolkit measures, Phoneme_LgFreq and Grapheme_LgFreq. This order of entry is arguably a conservative test of the value of the consistency measures, as it is not clear that lexical variables impinge upon outcome outcomes such as naming latency prior to sublexical variables. Nonetheless, these variables were entered first, as the focus is on determining the *unique* contribution of the various toolkit measures to explaining variance in the ELP behavioral data.
- *Step two:* The Phonographeme_LgFreq measure was entered second, on the basis that graphemic parsing and phonemic assignment occur early in the reading process (see Fig. 1). This measure does not reflect consistency and as such is not specific to either the reading or spelling direction.
- *Step three:* Phonographeme p(P|G) reading consistency was entered third, given the hypothesis that consistency at lower-level units takes precedence over high levels, although a reasonable case could be made for the reverse.
- *Step four:* Onset/Rime p(P|G) reading consistency was entered fourth, reflecting the higher-level nature of those units.
- *Step five:* Phonographeme p(G|P) spelling consistency was entered fifth, considering that feedback from phonological processing should arise only after feedforward processing begins.
- *Step six:* Onset/Rime p(G|P) spelling consistency was entered last, again considering that feedback and higher-level units might be expected to affect behavior last.

Table 2 reports the beta coefficients, associated *p* values, and the change in adjusted *R*^2^ (∆*R*^2^) for each variable when first entered in its respective stepwise model (e.g., the reported beta coefficient for Phonographeme p(P|G) reflects the magnitude and direction of its effect on the outcome measure when entered in step 3, not its value in the final model of step 6). We also report, in Table 3, the Bayesian information criterion (BIC) for each stepwise model; the BIC reflects a more conservative approach to identifying the best model, as it applies a greater penalty for model complexity and thus will tend to select a model with fewer variables. We adopt the common interpretation that a change in BIC (∆BIC) < 2 provides essentially no support, ∆BIC of 4–7 provides considerable support, and ∆BIC > 10 provides substantial support for the more complex model relative to the less complex one (Burnham & Anderson, 2004). The regression models, *R*^2^, and BIC values were all computed in R with the base *stats* package (version 4.2.1; R Core Team, 2022). Significance of the ∆*R*^2^ was assessed with *F*-statistics provided by the *anova* function in the *stats* package.

**Table 2:** Results of the stepwise regression of English Lexicon Project (ELP) Naming and Lexical Decision data, both reaction time (RT) and accuracy. *Bolded values* refer to *R*^2^ values, all other values refer to beta coefficients. *** *p* < 0.001, ** *p* < 0.01, * *p* < 0.05, ~ *p* < 0.10. RT = reaction time

	Naming		Lexical Decision	
	RT	Accuracy	RT	Accuracy
*Surface and lexical variables*				
Length	0.0455***	–0.0013	0.0108***	0.0083***
Ortho_N	– 0.0030**	0.0006*	0.0027**	0.0001
Phono_N	– 0.0005	– 0.0003*	0.0017***	–0.0008**
Freq_N	0.0001	0.0000	0.0028**	– 0.0008~
Freq_N_P	0.0005	– 0.0003	0.0034***	– 0.0008~
OLD	0.0425***	0.0003	0.1002***	– 0.0281***
OLDF	0.0392***	– 0.0069***	0.0286***	–0.0175***
PLD	0.0418***	–0.0067***	0.0313***	– 0.0014
PLDF	0.0348***	– 0.0061***	0.0286***	– 0.0155***
NSyll	0.0387***	– 0.0116***	0.0706***	– 0.0149***
NMorph	– 0.0916***	0.0174***	– 0.0632***	0.0317***
Phoneme_LgFreq	0.2153***	– 0.0110*	– 0.0001	0.0132
Grapheme_LgFreq	– 0.1526***	0.0252***	– 0.0543***	0.0174*
LgSUBTLWF	– 0.1160***	0.0219***	– 0.1766***	0.0711***
**Adjusted *R***^**2**^	**0.4095**	**0.1765**	**0.5125**	**0.3338**
*Consistency variables*				
Phonographeme_LgFreq	– 0.0757***	0.0199***	– 0.0312***	0.0160***
interaction with LgSUBTLWF	0.0086***	– 0.0049***	– 0.0041*	– 0.0024**
*Adjusted R*^*2*^	*0.4209*	*0.2024*	*0.5145*	*0.3365*
**∆*R***^***2***^	0.0114***	0.0259***	0.0021***	0.0028***
Phonographeme p(P|G)	– 0.0054	0.0041**	– 0.0135*	0.0052~
interaction with LgSUBTLWF	0.0225***	– 0.0043***	0.0028	0.0021*
*Adjusted R*^*2*^	*0.4270*	*0.2083*	*0.5148*	*0.3369*
**∆*R***^***2***^	0.0061***	0.0059***	0.0003*	0.0004*
Onset/Rime p(P|G)	– 0.0354***	0.0058***	– 0.0122***	0.0056***
interaction with LgSUBTLWF	0.0180***	– 0.0034***	0.0006	– 0.0025~
*Adjusted R*^*2*^	*0.4352*	*0.2140*	*0.5155*	*0.3379*
**∆*R***^***2***^	0.0082***	0.0057***	0.0007***	0.0009***
Phonographeme p(G|P)	0.0318***	– 0.0087***	0.0323***	– 0.0075**
interaction with LgSUBTLWF	0.0238***	– 0.0040***	0.0063*	0.0007
*Adjusted R*^*2*^	*0.4411*	*0.2206*	*0.5178*	*0.3384*
**∆*R***^***2***^	0.0059***	0.0066***	0.0023***	0.0005*
Onset/Rime p(G|P)	– 0.0086*	0.0001	– 0.0006	0.0010
interaction with LgSUBTLWF	0.0097**	– 0.0018*	0.0045	– 0.0019
*Adjusted R*^*2*^	*0.4417*	*0.2209*	*0.5178*	*0.3383*
***∆R***^***2***^	0.0007**	**0.0003~**	**0.00001**	**– 0.00002**

**Table 3:** BIC and ∆BIC for each regression model. ∆BIC < 2 provides essentially no support, ∆BIC of 4–7 provides considerable support, and ∆BIC > 10 provides substantial support for the more complex model relative to the less complex one. RT = reaction time

	Naming				Lexical decision			
	RT		Accuracy		RT		Accuracy	
Model	BIC	**∆BIC**	BIC	**∆BIC**	BIC	**∆BIC**	BIC	**∆BIC**
Surface and Lexical Variables	– 575		– 26899		– 1943		– 14800	
+ Phonographeme_LgFreq	– 728	**– 153**	– 27073	**– 175**	– 1970	**– 27**	– 14824	**– 24**
*× LgSUBTLWF*	– 738	**– 10**	– 27175	**– 102**	– 1966	**4**	– 14822	**2**
+ Phonographeme p(P|G)	– 729	**9**	– 27173	**2**	– 1962	**3**	– 14816	**5**
*× LgSUBTLWF*	– 819	**– 89**	– 27227	**– 54**	– 1955	**7**	– 14811	**5**
+ Onset/Rime p(P|G)	– 901	**– 82**	– 27259	**– 32**	– 1961	**– 6**	– 14813	**– 2**
*× LgSUBTLWF*	– 934	**– 33**	– 27276	**– 17**	– 1952	**9**	– 14808	**5**
+ Phonographeme p(G|P)	– 963	**– 29**	– 27317	**– 40**	– 1984	**– 32**	– 14807	**0**
*× LgSUBTLWF*	– 1014	**– 51**	– 27338	**– 21**	– 1979	**4**	– 14798	**9**
+ Onset/Rime p(G|P)	– 1009	**5**	– 27329	**9**	– 1970	**9**	– 14789	**9**
*× LgSUBTLWF*	– 1009	**0**	– 27325	**4**	– 1963	**7**	– 14782	**8**

### Stepwise regression results

- *Step one:* As shown in Table 2, the surface and lexical variables entered together in step one explained significant variance: total *R*^2^ = 0.41 for Naming RT, 0.18 for Naming Accuracy, 0.51 for Lexical Decision RT, and 0.33 for Lexical Decision Accuracy. With respect to the toolkit measures in this base model, first of all Phoneme_LgFreq was a significant predictor of both Naming RT and Accuracy such that words with more frequent phonemes were read more slowly (*p* < 0.001) and less accurately (*p* < 0.05) relative to words with less frequent phonemes. Because the direction of these effects is opposite what one might expect, we examined the first-order correlations between Phoneme_LgFreq and Naming RT and Accuracy to determine whether there might be a suppression effect in the multiple regression. This revealed that the association with RT was positive even without controlling for the other variables in step one, whereas the association with Accuracy was indeed reversed^6^. There was no significant relationship between Phoneme_LgFreq and Lexical Decision RT or Accuracy.
- Second, Grapheme_LgFreq was a significant predictor of all four outcomes (*p*’s < 0.05), such that responses were both faster and more accurate for words with relatively more frequent graphemes.
- *Step two:* The effect of Phonographeme_LgFreq was significant for all four outcome measures (*p*’s < 0.001), as were the interactions with word frequency. For both Naming and Lexical Decision, words with frequent phonographemes were responded to more quickly and more accurately. This effect was attenuated for high- compared to low-frequency words for Naming RT, Naming accuracy, and Lexical Decision accuracy. The reverse was true of Lexical Decision RT (the effect was attenuated for low- compared to high-frequency words), although this *p* value was less robust (*p* ≈ 03, compared to *p* ≈ 0.009 for Lexical Decision accuracy and < 0.001 for Naming RT and accuracy). The ∆R^2^ was significant for all four outcome measures, explaining an additional 1.14% for Naming RT, 2.49% for Naming Accuracy, 0.21% for Lexical Decision RT, and 0.28% for Lexical Decision Accuracy.
- *Step three:* The effect of Phonographeme p(P|G) consistency was significant for Naming accuracy (*p* < 0.01) and for Lexical Decision RT (*p* < 0.05). While it was not significant for Naming RT or Lexical Decision accuracy, the interactions with lexical frequency were significant for all but Lexical Decision RT (*p*’s < 0.05). Specifically, For Naming, words with higher reading consistency were read more quickly and more accurately, and these effects were significantly greater for low-frequency compared to high-frequency words. For Lexical Decision, words with higher reading consistency were responded to more quickly and more accurately, but in the case of accuracy the effect was greater for high- compared to low-frequency words. The ∆*R*^2^ was significant for all four outcome measures (*p*’s < 0.001), explaining an additional 0.61% for Naming RT, 0.59% for Naming Accuracy, 0.03% for Lexical Decision RT, and 0.04% for Lexical Decision Accuracy.
- *Step four:* The effect of Onset/Rime p(P|G) consistency was significant for all four outcome measures (*p*’s < 0.001), with responses being both faster and more accurate for words with greater reading consistency. The interaction with word frequency was significant for both Naming outcomes (*p*’s < 0.001) but neither of the Lexical Decision outcomes (*p*’s > 0.05). For Naming, the effects of onset/rime reading consistency were greater for low- compared to high-frequency words. The ∆*R*^2^ was significant for all four outcome measures (*p*’s < 0.001), explaining an additional 0.82% for Naming RT, 0.57% for Naming Accuracy, 0.07% for Lexical Decision RT, and 0.09% for Lexical Decision Accuracy.
- *Step five:* The effect of Phonographeme p(G|P) spelling consistency was significant for all four outcome measures (*p*’s < 0.001 except for Lexical Decision Accuracy, *p* < 0.01). The interaction with word frequency was significant for both Naming outcomes (*p*’s < 0.001) and for Lexical Decision RT (*p* < 0.05) but not Lexical Decision Accuracy (*p* < 0.10). Interestingly, for Naming, words with higher spelling consistency were read more *slowly* and *less* accurately, and these effects were significantly greater for high-frequency compared to low-frequency words. For Lexical Decision, as with Naming words with higher spelling consistency were responded to more slowly and less accurately, and this effect on RT was again greater for high- compared to low-frequency words. These findings may indicate interference/competition due to feedback. The ∆*R*^2^ was significant for all four outcome measures (*p*’s < 0.001 except for Lexical Decision Accuracy, *p* < 0.05), explaining an additional 0.59% for Naming RT, 0.66% for Naming Accuracy, 0.23% for Lexical Decision RT, and 0.05% for Lexical Decision Accuracy.
- *Step six:* The effect of feedback Onset/Rime p(G|P) spelling consistency was significant only for Naming RT (*p* < 0.05), and the interaction with word frequency was significant for both Naming RT (*p* < 0.01) and Naming Accuracy (*p* < 0.01); no significant effects were found for Lexical Decision RT (*p*’s > 0.10). For Naming RT, words with consistent mappings were read more quickly, and this effect was significantly greater for low-frequency compared to high-frequency words. For Naming Accuracy, the significant interaction of consistency by word frequency indicated that low-frequency words relative to high-frequency words were read more accurately if the mapping was consistent. The ∆*R*^2^ was significant only for Naming RT (*p* < 0.01) and marginally so for accuracy (*p* < 0.10), explaining an additional 0.07% for Naming RT and 0.03% for accuracy.

The BIC values are reported in Table 3 for each stepwise regression, reporting main effects prior to interactions. Negative ∆BIC indicates support in favor of the more complex model and positive ∆BIC indicates support against the more complex model. *Naming RT*: all but two measures have substantial support for improving model fit. The first exception is Phonographeme p(P|G), which has considerable support *against* being included, however there is substantial support in favor of its interaction with word frequency. This suggests that reading consistency’s effects on Naming are particularly tied to lexical frequency (e.g., only low-frequency words benefit from having consistent mappings). Second, there is moderate evidence *against* including Onset/Rime p(P|G), and no evidence in support of it interacting with word frequency. *Naming Accuracy*: the pattern of results mirrors that for Naming RT. *Lexical Decision RT*: only Phonographeme_LgFreq, Onset/Rime p(P|G) reading consistency, and Phonographeme p(G|P) spelling consistency receive considerable or substantial support. There is considerable support *against* including any of the interactions with word frequency as well as against Onset/Rime p(G|P) spelling consistency. *Lexical Decision Accuracy*: there is substantial support only for Phonographeme_LgFreq, and considerable support *against* most of the other measures (no evidence either way for Phonographeme_LgFreq interacting with word frequency, Onset/Rime p(P|G), or Phonographeme p(G|P)) .

### Stepwise regression summary

The results of the stepwise regression analyses in Tables 2 and 3 confirm that the toolkit measures explain significant amounts of unique variance after controlling for surface and lexical variables. The directions of the effects are *generally* as expected, with greater consistency/frequency associated with faster and more accurate responses. Similarly, interactions with word frequency typically indicate greater effects for low-frequency relative to high-frequency words. However, in some instances the effects of p(G|P) spelling consistency, which can be considered as feedback in the context of reading tasks, were opposite those of p(P)G) reading consistency. The pattern of reverse effects for feedback relative to feedforward consistency was also observed in some of the analyses of ELP data conducted by Chee et al., (2020), and previously observed in Yap and Balota (2009). In those studies, the apparently inhibitory effects arising from feedback were linked specifically to onset consistency; here, we did not separately model onset and rime consistency but rather only the composite of the two. Instead, here the inhibitory effects were found for Phonographeme p(G|P) spelling consistency, which may or may not be driven by the onsets in particular.

The BIC analyses (Table 3) present a more parsimonious account, in particular suggesting a much smaller role for consistency in Lexical Decision compared to Naming, and then only for consistency at the phonographeme level, not onset/rime. The BIC approach indicates that the difficult-to-interpret interaction between phonographeme reading consistency and word frequency in the context of Lexical Decision RT (Table 2, *p* ≈ 0.03) is quite possibly spurious, as the evidence goes *against* it (ΔBIC 7). It also presents substantial evidence against the possibility that feedback from the level of the onset/rime p(G|P) plays a role in either Naming or Lexical Decision, although these results might differ if the stepwise procedure entered variables in a different order.

### Elastic net regression analyses

To address the possibility that the selected order of variables biases the results presented here, we also conducted an elastic net regression. This approach provides a method of determining the relative importance of the variables in an unconstrained fashion (i.e., without specifying an order of entry as in stepwise regression). Elastic net regression (Zou & Hastie, 2005) combines the benefits of the least absolute shrinkage and selection operator (LASSO) method, which supports variable selection by setting the coefficients of less important ones to zero (Tibshirani, 2011), and ridge regression, which outperforms LASSO regression in the case of highly correlated variables (Hastie, Tibshirani, & Friedman, 2009). Importantly, this has an effect of protecting “the estimates for the coefficients against collinearity-induced enhancement” (page 263; Tomaschek et al., 2018), essentially taking into account correlations between the predictors.

**Fig. 3:**
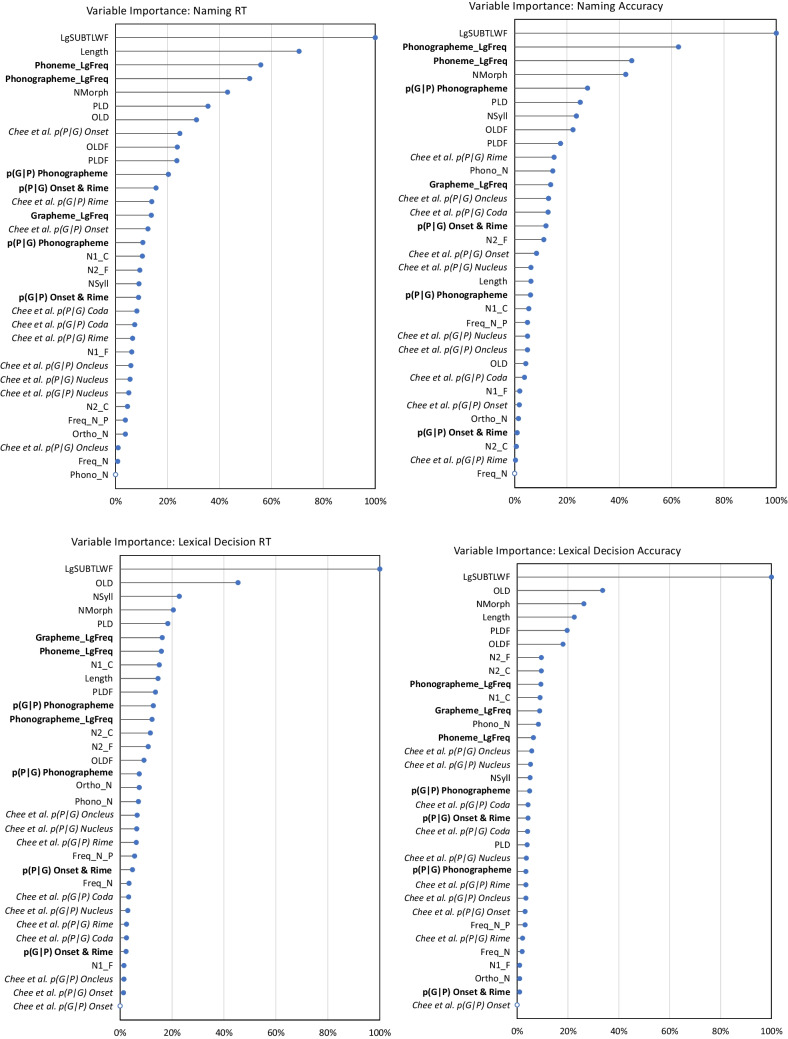
Variable importance from elastic net regressions. Toolkit measures in *bold*, consistency measures from Chee et al. (2020) in *italics*. RT = reaction time

All of the surface, lexical, and toolkit measures included in the stepwise regression models were included in the elastic net regression, as well as the ten consistency measures from Chee et al. (2020): Onset, Nucleus, Coda, Oncleus, and Rime consistency in both the reading and spelling directions. The R package *glmnet* (version 4.1-4; Friedman et al., 2010) was used to fit the elastic net regression, and variable importance was obtained from the package *caret* (version 6.0-93; Kuhn, 2022), function *varImp*. Repeated tenfold cross-validation was used with 20 repetitions, separately for each of the four outcome measures: Naming RT, Naming Accuracy, Lexical Decision RT, and Lexical Decision Accuracy. The primary results of interest are the variable importance metrics, reflecting the absolute value of the scaled coefficients. Specifically, all variables were *Z*-scored prior to entry in the elastic net regression, and Fig. 3 depicts the variable importance as relative to the predictor with the largest coefficient (which was word frequency, for all outcome measures).

### Elastic net regression results

Figure 3 reports variable importance relative to the predictor with the largest coefficient, which was word frequency for all outcome measures. *Naming RT (*Fig. 3*, top left):* Of the 14 consistency measures (ten from Chee et al., 2020, and four from the toolkits), the most important was Chee and colleagues’ Onset reading consistency (25% as important as word frequency, ranked 8^th^), followed by the Sublexical Toolkit’s Phonographeme spelling consistency (20%, ranked 11^th^) and Onset/Rime reading consistency (16%, ranked 12^th^). However, the toolkit’s Phonographeme_LgFreq measure was more important than any of the consistency measures (52%, ranked 4^th^), as was its measure of phoneme frequency (Phoneme_LgFreq, 56%, ranked 3^rd^). The only consistency measure with importance less than 1% of that of word frequency was Chee and colleagues’ Oncleus reading consistency (1%, ranked 31^st^).

### Naming Accuracy (Fig. 3, top right)

The most important consistency measure was the toolkit’s Phonographeme spelling consistency (28%, ranked 5^th^), followed by Chee and colleagues’ Rime reading consistency (15%, 10^th^) and Oncleus reading consistency (13%, ranked 13^th^). However, as with Naming RT, both the toolkit’s Phonographeme_LgFreq (63%, ranked 2^nd^) and Phoneme_LgFreq (45%, ranked 3^rd^) measures from the were more important. Two consistency measures had less than 1% of the importance of word frequency: Chee and colleagues’ Rime spelling consistency (0.3%, ranked 32^nd^) and the toolkit’s Onset/Rime spelling consistency (1%, ranked 30^th^).

### Lexical Decision RT (Fig. 3, bottom left)

The most important consistency measure was the toolkit’s Phonographeme spelling consistency (13%, ranked 11^th^), followed by its Phonographeme reading consistency (7%, 16^th^) and Chee and colleagues’ Oncleus reading consistency (7%, ranked 19^th^). Both the toolkit’s Grapheme_LgFreq (16%, ranked 6^th^) and Phoneme_LgFreq (16%, ranked 7^th^) measures were more important. The only consistency measure with importance less than 1% of that of word frequency was Chee and colleagues’ Onset spelling consistency (0%, ranked 33^rd^).

### Lexical Decision Accuracy (Fig. 3, bottom right)

The most important consistency measure was Chee and colleagues’ Oncleus reading consistency (6%, ranked 14^th^), followed by their Nucleus spelling consistency (5%, 15^th^) and the Sublexical Toolkit’s Phonographeme spelling consistency measure (5%, ranked 17^th^). However, the three frequency measures from the Sublexical Toolkit all ranked as more important: Phonographeme_LgFreq (9%, ranked 9^th^), Grapheme_LgFreq (9%, ranked 11^th^), and Phoneme_LgFreq (6%, ranked 13^th^). Two consistency measures had less than 1% of the importance of word frequency: Chee and colleagues’ Onset spelling consistency (0%, ranked 33^rd^) and the toolkit’s Onset/Rime spelling consistency (1%, ranked 32^nd^).

### Elastic net regression summary

The elastic net regression was used as a data-driven approach to assess the relative importance of the various predictors, and thus compliments the findings from the stepwise regression by adopting a more theory-neutral stance. With word frequency being the single most important variable for all four outcome measures, it is possible to use that measure’s importance as a “benchmark” against which to judge the others. In that respect, several measures from the toolkit performed very well: looking across all four outcome measures, only five variables approached or exceeded 50% of the importance of word frequency. Of those five, three were from the toolkit: Phoneme_LgFreq for Naming RT and Phonographeme_LgFreq for both Naming RT and Accuracy (the other two were word length for Naming RT and OLD, the average Levenshtein distance from the 20 closest neighbor words, for Lexical Decision RT).

Whereas in the stepwise regression phonographeme frequency was forced in as the first measure after surface and lexical variables (i.e., in step two), its importance in the elastic net regressions was not determined a priori. Nonetheless, it emerged as more important than any of the consistency measures, including those from Chee and colleagues, except in the case of Lexical Decision RT (where it was nearly tied with the Sublexical Toolkit’s Phonographeme spelling consistency measure). This is further validation that the phonographemes are important units of sublexical representation and suggests the possibility that their frequency may be more important than their consistency.

Although not the focus of this work, the phoneme and grapheme frequency measures from the Sublexical Toolkit were also among the most important variables, outperforming not only the consistency measures but also most of the surface and lexical variables. In particular, grapheme frequency outranked all of the unigram and bigram measures (N1_F, N1_C, N2_F, and N2_C) for Naming RT, Naming Accuracy, and Lexical Decision RT (although not Lexical Decision Accuracy). This may be an indication that graphemes are a more important representational unit than single letters or bigrams, but it may also be attributable to the both-ends position coding scheme used in the toolkit (whereas standard unigram and bigram measures are based on serial position in the word).

Similar to the results of the stepwise regression, the consistency measures were generally more important for predicting Naming (both RT and accuracy) than Lexical Decision, and feedback (spelling consistency) measures were generally less important than feedforward (reading consistency) measures. This was particularly true for the higher-level units such as rimes, also consistent with the findings of the stepwise regression. For example, adding the “feedback” Onset/Rime p(G|P) measure resulted in *worse* model fits per BIC values (see Table 3).

## Accounting for variability in pseudoword reading

A final test of the toolkit measures’ validity assesses their ability to account for variance in pseudoword reading at the population level, i.e., to account for the distribution of alternative pronunciations of pseudowords across a group of participants presented with the same words. Recently, Coltheart and Ulicheva (2018) addressed the question of the source of variability in the pronunciation of pseudowords in adult skilled readers of English and identified two factors: differences in graphemic parsing and differences in phonemic assignment. It is the latter of these factors that the toolkit is well equipped to address.

The analyses of Coltheart and Ulicheva (2018) focused on variability both within-items (across participants) and within-participants (across items) in what amounts to the level of phonographeme units. In addition to revealing that the same written stimulus may be *parsed* into different graphemes (e.g., BLUISE parsed by some participants as one syllable, others as two syllables BLU-ISE, and still others as three syllables BLU-I-SE), they also found that the same graphemic parsing could result in different pronunciations due to alternative phonemic assignment (e.g., monosyllabic BLUISE read /bluz/ or /blus/). This phenomenon was quantified by calculating a measure of entropy, both for each grapheme and for each participant. For example, the grapheme [B]’s entropy was nearly zero, reflecting that in almost all instances (across words and participants) it was mapped to the phoneme /b/. However, for the grapheme [Y] entropy was very high, reflecting its being mapped alternatively to the phonemes /i/, /j/, /ɪ/ or others, depending on the context (the word and/or the participant). These measures were not computed with respect to specific positions of graphemes within words, nor were they computed for larger units such as rimes.

To the best of our knowledge, no previous work has attempted to account for the distribution of *specific* pseudoword reading responses across participants. While some previous efforts have investigated factors which contribute to pseudoword naming, such as the influence of surrounding consonants on vowel pronunciation, the statistical analyses have only compared “correct” and “incorrect” responses (e.g., Steacy et al., 2019) or proportions of pronunciations that met some pre-defined criteria (such as choosing a particular vowel given specific surrounding contexts; Treiman et al., 2003, 2006; Treiman & Kessler, 2023). Ulicheva and colleagues (Ulicheva et al., 2021) presented a set of 50 pseudowords to participants to read repeatedly across multiple sessions on different days and assessed the stability of responses using measures of entropy. The authors reported that words whose graphemes afford multiple potential readings were pronounced with more variability from session to session (e.g., BUDGORD had 15 unique pronunciations and therefore high entropy), compared to words whose graphemes afford fewer opportunities for such variability (e.g., MISCLEAF, just two unique pronunciations and therefore low entropy). Their quantification of the potential number of readings (i.e., the number of plausible grapheme-phoneme mappings) explained a significant, but very small, amount of the total session-to-session variance in responses (*R*^2^ ≈ 2%).

**Fig. 4:**
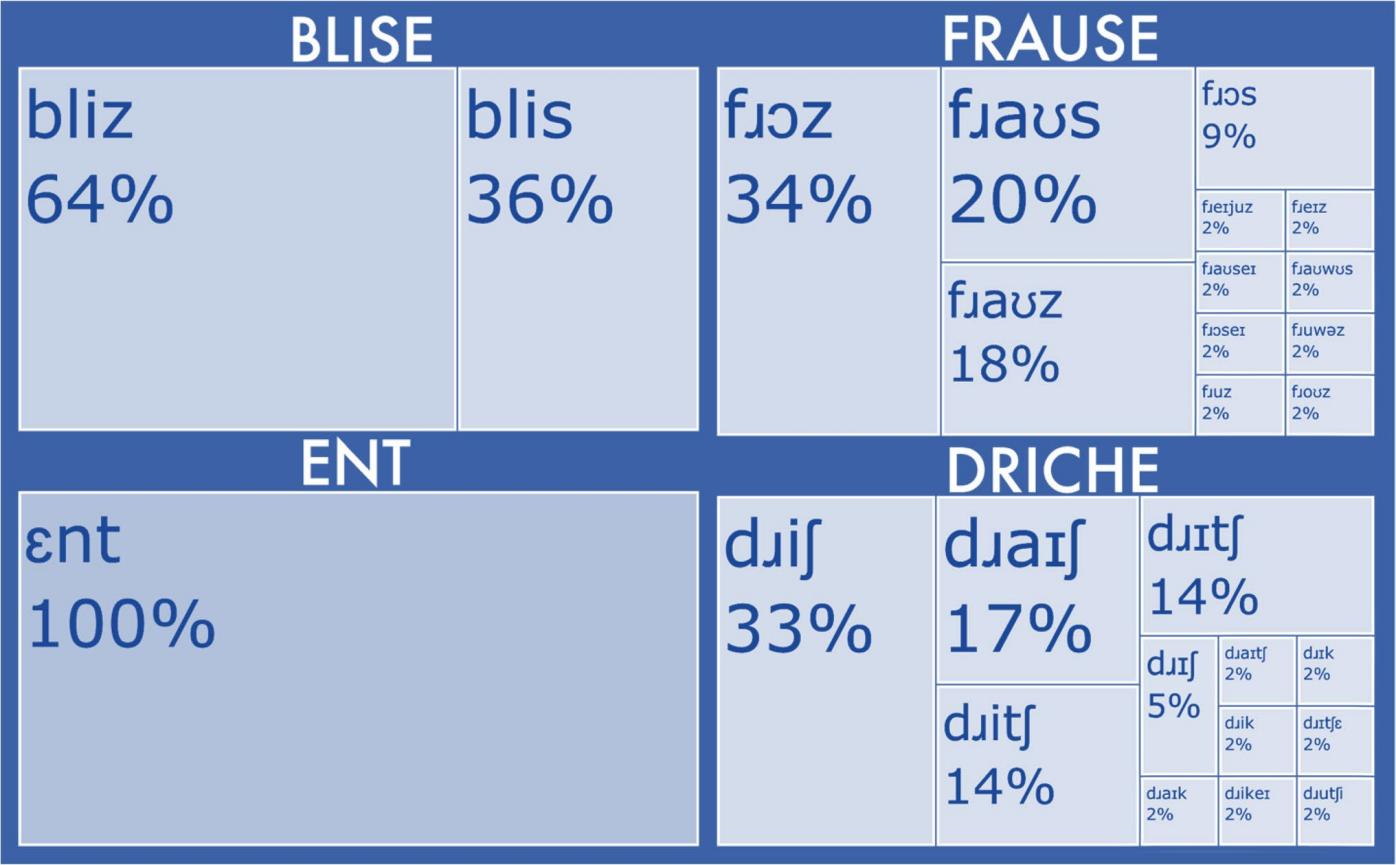
Examples of various pseudoword pronunciations and their relative frequencies

To the best of our knowledge, we provide the first attempt to account for the *specific* pronunciations that participants generate during reading (but see Authors, 2023, for an application of an early version of the toolkit to explaining specific written responses in the context of pseudoword spelling to dictation). Crucially, this approach does not entail judgments of accuracy, and allows for differentiating between responses that would be considered “correct” but nonetheless differ in their prominence (as in BLISE read either /blis/ or /bliz/). Here, we do not seek to account for stability of pronunciations across time, nor a measure of entropy that collapses the distribution of unique responses into a single measure. Instead, we use Poisson regression to model the counts of different pronunciations elicited for a large set of items presented in a pseudoword reading task. For example (Fig. 4), the word BLEASE was read as /bliz/ by 64% of participants and /blis/ by 36%; FRAUSE was read as /fɹɔz/ by 34%, /fɹaʊs/ by 20%, /fɹeɪ juz/ by 2%, etc. Given that the vast majority of English graphemes correspond to multiple possible phonemes across the English lexicon, we predict that the more common pronunciations should be associated with more consistent mappings. This prediction stems from the characterization of the sublexical system depicted in Figs. 1 and 2. Importantly, these measures are continuous, and so support capturing not only which pronunciation will be most prevalent, but also the rate at which uncommon-but-plausible pronunciations may be observed.

### Materials

The pseudowords and responses were taken from the publicly available dataset of Pritchard et al. (2012), which were also used in Coltheart & Ulicheva (2018). In total, 412 words were presented to 45 speakers who read each word aloud. The transcription of the pronunciations was carried out by the original authors (Pritchard and colleagues), but for our purposes these were re-transcribed to account for differences between Australian English (as the original dataset was collected in Australia) and American English. While there are a number of differences between the two, the one that must necessarily be addressed in order to apply the toolkit regards rhotic vowels (“r-colored” vowels). Specifically, the non-rhotic pronunciations of the Australian participants were re-transcribed as rhotic, simply by inserting an /ɹ/ after the vowel. This is necessary for the toolkit because it is based on rhotic American accents and consequently requires that [R] graphemes correspond to some phoneme. For example, BROR was read as /bɹɔ/ by nearly all participants, with no phoneme corresponding to the second R. Had the toolkits been developed based on non-rhotic English, the mapping would have been OR ➔ /ɔ/. Instead, to fit the American English mappings, the pronunciation was re-transcribed as /bɹɔɹ/, so that the second R ➔ /ɹ/. This approach was used for all instances where an orthographic R (or RR, or WR, etc.) did not correspond to any phoneme in the Australian pronunciation.

Across the 412 words, 3233 unique pronunciations were elicited (mean = 7.85 unique pronunciations per word, range 1–23). Of these, 67 words were excluded because a measure was not possible for either the onset or the rime (either because they do not exist in any real English word, are so uncommon that they have not been coded in the toolkit corpus, or simply did not exist in the case of the onsets [e.g., the word OOSH has no onset]). That left 345 words with 2634 unique pronunciations. The counts of these pronunciations were taken as the dependent measure and modeled using generalized mixed-effects regression with the Poisson distribution, using the R package *glmmTMB* (version 1.1.5, Brooks et al., 2017). The total counts per word ranged somewhat, from 39 to 45, because some participants did not provide responses to every single word (see Pritchard et al., 2012). The counts per pronunciation ranged from 1 to 45 (proportionally, from 2.22% to 100%).

A number of variables were included as predictors that might plausibly be associated with the rate at which the various pronunciations were observed. As with the regression analyses of ELP data, these 14 variables may be categorized as surface, lexical, or sublexical. *Surface variables:* Phoneme frequency and Phonotactics (i.e., uniphone and biphone frequency), taken from Vitevich & Luce (2004), number of syllables (as a measure of length as well as graphemic parsing), and Phoneme_LgFreq from the Sublexical Toolkit. Note that purely orthographic measures, such as bigram frequency or the toolkit’s Grapheme_LgFreq, were not included because by definition they do not differentiate between alternative spoken productions (e.g., bigram frequency cannot contribute to explaining why more people pronounce BLISE as /bliz/ than /blis/). *Lexical variables:* lexical status (whether or not the pseudoword was pronounced as a real word, e.g., BLISE read as BLISS), PTAN (number of phonological neighbors), and PGTAN (number of phonographic neighbors). PTAN was retrieved from the CLEARPOND database (Marian et al., 2012), and PGTAN was calculated manually by counting the number of shared phonological and orthographic neighbors as indicated by CLEARPOND. *Sublexical variables:* Phonographeme_LgFreq, and six consistency measures from the toolkit – both p(P|G) reading consistency and p(G|P) spelling consistency at the level of phonographemes, onsets, and rimes (onsets and rimes measured separately rather than as a composite score). It should be emphasized that other published consistency measures are not readily able to generate consistency measures for pseudowords, and thus only the toolkit consistency measures were included in the regression.

Whereas the analyses presented up to this point were constrained to using the mean across all segments^7^, this one is free to explore alternatives to the mean. Therefore, for the phoneme frequency, phonotactic, and sublexical measures, both the mean *and* the minimum across all segments were tested in separate models in order to determine which explained the most variance in behavior. The total model *R*^2^ was higher when using the minimum as the statistic compared to the mean, and therefore only that model is reported here (the pattern of results was similar but weaker when using the mean as the statistic). Notably, the use of the minimum across all segments was also observed to explain the most variance in pseudoword spelling behavior in a recent study using the toolkit (Authors, 2023). Multicollinearity was first assessed when entering all of the variables simultaneously, which revealed VIF > 10 for the two Onset consistency measures and > 6 for Phonographeme_LgFreq. Onset spelling consistency was removed, considering that the reading consistency measure is more important for reading, and doing so reduced the VIF to just 1.3. Phoneme_LgFreq was also removed in consideration that it is conceptually similar to the phoneme frequency measure from Vitevitch & Luce (2004), which reduced the Phonographeme_LgFreq VIF to 3.7. All remaining VIF’s were < 6.

In addition to fixed effects for the 12 remaining predictors, random effects were included in the form of a random intercept by-pronunciations nested in words. This random effect significantly improved model fit compared to a fixed effects only model, ∆BIC -4754, log ratio test *p* value < 0.001. In addition, the model was checked for overdispersion using the R package *performance* (version 0.9.2, Lüdecke et al., 2021), which was found not to be an issue (dispersion ratio = 0.218, Chi-squared = 572, *p* value ≈ 1.0).

Finally, *R*^2^ was computed using the R package *MuMIn* with the recommended “trigamma” estimate (version 1.47.1, Bartoń, 2022) for both the marginal effects (fixed effects only) and conditional effects (fixed+random effects). In addition to total *R*^2^ for the full model, each predictor was assessed for the maximum amount of variance it can explain by entering it first in the model (i.e., as the only predictor) as well as the amount of variance it uniquely explains (i.e., the change in *R*^2^ when adding that predictor last in the model).

### Pseudoword variability results

The results of the Poisson regression are reported in Table 4, with the first-in and unique variance explained by each predictor reported in Table 5. The model with all variables entered simultaneously had a total marginal *R*^2^ of 57.1% and conditional *R*^2^ of 87.5%. All predictors explained significant “unique variance” at *p* < 0.05 except for four: lexical status, phonotactics, PTAN, and Phonographeme spelling consistency. However, “first-in” variance explained was significant even for those predictors (marginally so in the case of lexical status).

**Table 4:** Results of the mixed-effects Poisson regression predicting variability in pseudoword responses. PTAN = number of phonological neighbors; PGTAN = number of phonographic neighbors; p(P|G) = reading consistency, p(G|P) = spelling consistency. *P* values based on Wald *Z*-value, *** *p* < 0.001, ** *p* < 0.01, * *p* < 0.05, ~ *p* < 0.10. *R*^2^ = pseudo *R*^2^, trigamma estimate (Bartoń, 2022); marginal = fixed effects only, conditional = fixed+random effects

Regressor	Estimate	Std. Error	Wald *Z*-value	*p* value
(Intercept)	– 2.880	0.031	– 91.77	< 2.00E-16***
Lexical Status	0.006	0.032	0.17	0.86227
Syllables	– 0.208	0.024	– 8.68	< 2.00E-16***
Phonemes	– 0.044	0.022	– 1.98	0.04788*
Phonotactics	0.036	0.023	1.59	0.11256
PTAN	– 0.037	0.023	– 1.6	0.10894
PGTAN	0.055	0.018	3.14	0.00167**
Phonographeme_LgFreq	0.217	0.035	6.19	5.95E-10***
Phonographeme p(P|G)	0.367	0.030	12.23	< 2.00E-16***
Onset p(P|G)	0.305	0.024	12.85	< 2.00E-16***
Rime p(P|G)	0.208	0.041	5.07	4.01E-07***
Phonographeme p(G|P)	0.056	0.033	1.73	0.08291~
Rime p(G|P)	0.174	0.040	4.35	1.37E-05***
	marginal	conditional		
*R*^2^	57.05%	87.50%

**Table 5:** Contributions to *R*^2^ for variables when added first to the model (first-in), and unique *R*^2^ contribution (last-in). PTAN = number of phonological neighbors; PGTAN = number of phonographic neighbors; p(P|G) = reading consistency, p(G|P) = spelling consistency. Total model *R*^2^ (marginal, i.e., fixed effects only) = 57.05%. *p* values based on Chi-squared test (log likelihood), *** *p* < 0.001, ** *p* < 0.01, * *p* < 0.05, ~ *p* < 0.10

Regressor	First-In *R*^2^	Unique *R*^2^
Phonographeme p(P|G)	41.11%***	1.811%***
Phonographeme_LgFreq	39.18%***	1.212%***
Phonographeme p(G|P)	32.41%***	0.019%~
Rime p(P|G)	20.40%***	0.558%***
Rime p(G|P)	19.62%***	0.334%***
Onset p(P|G)	14.52%***	3.715%***
Syllables	7.29%***	2.006%***
PGTAN	5.44%***	0.125%**
Phonotactics	1.46%***	0.052%
Phonemes	1.18%***	0.088%*
PTAN	0.52%***	0.073%
Lexical	0.16%~	– 0.004%

In terms of the amount of “first-in” or maximal variance explained (Table 5), the single most predictive regressor was Phonographeme reading consistency, which alone could account for 41.1% of the variance. This was followed closely by Phonographeme_LgFreq (39.2%). Critically, the top six variables were all sublexical from the toolkit; the best surface variable was number of syllables (7.3%) and the best lexical variable was PGTAN (5.4%).

As for “unique” variance explained, although Phonographeme reading consistency was the most predictive measure when entered first, it was only ranked third when entered last (unique *R*^2^ = 1.8%). The greatest amount of *unique* variance explained was attributed to Onset reading consistency (3.7%), followed by the number of syllables (2.0%). Interestingly, although the first-in variance explained by feedback Phonographeme spelling consistency was very high (32.4%), virtually all of this variance was shared with other variables, reflected in its small, non-significant unique variance explained (just 0.02%, *p* < 0.10).

### Pseudoword Variability Summary

By coding in the toolkit each of the alternative pronunciations generated by the sample of human participants, it was possible to confirm that the most frequently observed pronunciations tended to have higher consistency. In fact, all of the significant lexical and sublexical predictors showed positive associations^8^: the most common pronunciations were both more consistent and had more lexical neighbors. The regression explained the majority of the variance in the counts (57.1% from the fixed effects alone, 87.5% including the random effects), and all of the most important predictors were those from the toolkit. The single most powerful predictor was Phonographeme reading consistency (*R*^2^ = 41.1%), while the predictor with the greatest *unique* variance explained was Onset reading consistency (3.7%). Importantly, all of the consistency measures were much better predictors than any of the other surface or lexical measures considered, with only three of those contributing any amount of unique variance (the number of syllables, the number of phonographic neighbors, and the phoneme frequency measure of Vitevitch & Luce, 2004).

These results further demonstrate how the Sublexical Toolkit supports conducting novel analyses of reading and spelling data, in particular because it is readily applicable to pseudowords. Parallel to the analyses of the ELP data (both oral naming and lexical decision), the simultaneous regression supports the hypothesis that sublexical processes integrate information across levels of representation (phonographeme and onset/rime). However, due to the nature of these data, it is possible that this result is driven by individual differences (some participants generate pronunciations from the phonographeme level, others from the onset/rime level) or item effects (some items are explained by rime processing, others are not), and not necessarily integration of levels of processing within-item and within-individuals.

In the specific context of pseudoword reading, there was also evidence that “feedback” processes also influenced how individuals pronounced the words, potentially adjusting their responses to improve their spelling consistency – although it should be noted that the variance uniquely explained by the feedback measures was relatively small. Finally, the results also confirm that the toolkit has validity even when applied to data from speakers of a non-American variant of English (here, Australian), requiring only modest adaptations (in particular, an /ɹ/ ➔ R mapping had to be inserted to account for the non-rhoticity of the Australian accent).

## General discussion

**Fig. 5:**
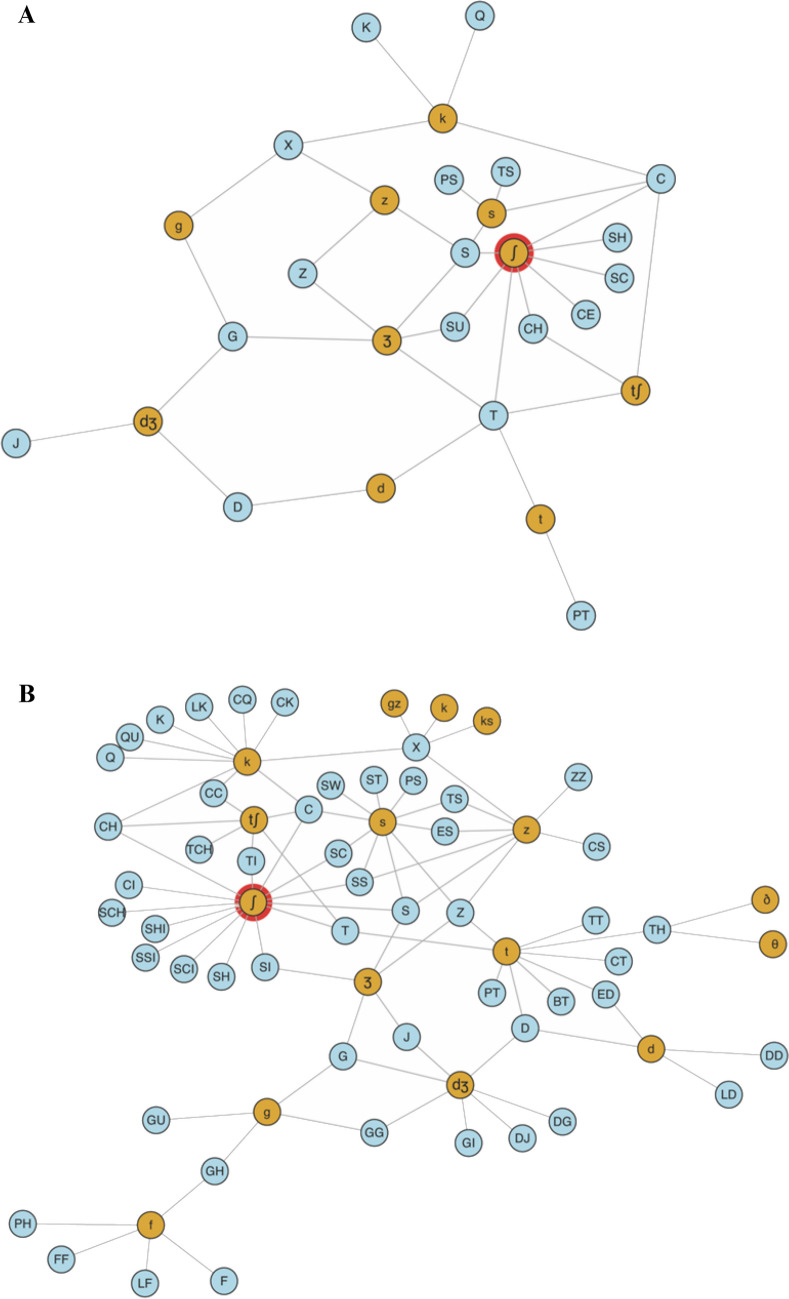
A representation of the sublexical system as nodes-and-links between phonemes (*yellow*) and graphemes (*blue*). **A** The graphemic inventory of Chee et al. (2020); **B** that of the current work. The networks here depict the connections from the phoneme /ʃ/ (*circled in red*) to all of its potential graphemes, as well as between each of those graphemes to the phonemes they represent, and so on until no other potential mappings remaining. As such, this network represents a phonographemic “island” of interrelated phonemes and graphemes. The more exhaustive inventory of graphemes in the current work results in greater network complexity (panel B compared to panel A)

The work presented here operationalizes the extraction of measures of spelling-sound regularities from the English lexicon in a novel way. It conceptualizes sublexical processing as a system that, via accumulated experience with associating spoken and written word forms, develops a network of interconnected phonemes and graphemes of different grain sizes (Fig. 5). Crucially, a central assumption of this work is that the sublexical system is a productive one that allows individuals to generate spellings or pronunciations for novel words, and as such is critical for self-teaching (e.g., Share, 1995). This perspective entails a bottom-up approach to measuring spelling-sound consistency, as opposed to traditional perspectives that attempt to identify a finite set of “rules” that describe the majority of English spelling patterns. An advantage of the bottom-up approach is that no arbitrary distinctions are made between “regular” and “irregular” spellings; rather, all spellings exist on a continuum from relatively more or less consistent, determined simply by the frequency with which they are encountered in the lexicon.

The knowledge represented in the toolkit can be visualized as nodes-and-links between phonemes and graphemes, as in Fig. 5. Portraying the many-to-many sound–spelling mappings of English in this way makes it easier to understand the origin of “creative spellings” and the inherent difficulty of recalling some spellings. For example, the I in FISH could instead be spelled Y or I_E, the F could be PH, and the SH could be SCH or CH – leading to such alternates as FISHE, FISCH, or even PHYCHE (although note that the oft-cited GHOTI, e.g., Zimmer, 2010, actually violates positional constraints – GH is /f/ only in final positions, as in ROUGH, and TI is /ʃ/ only in non-final positions, as in LOTION). The figure depicts all of the graphemes connected to the phoneme /ʃ/ (highlighted in red), all of the phonemes connected to those graphemes, and so on, until an “island” of phoneme-grapheme connections is complete. The Sublexical Toolkit quantifies this network in multiple ways: the strengths of the nodes are measured by phoneme and grapheme frequency (e.g., the number of words with /ʃ/ in the initial position), and the strengths of the links are measured both without respect to direction, as phonographeme frequency (e.g., the number of words with /ʃ/ in the initial position spelled SH), and with respect to the spelling direction (e.g., the *proportion* of words with /ʃ/ in the initial position spelled SH) and reading direction (e.g., the *proportion* of words with SH in the initial position pronounced /ʃ/). It functions similarly at the onset/rime level, but with larger grain sizes (such as the onset /ʃl/ spelled SCHL or the rime /ɛp/ spelled EP).

We have demonstrated that these measures explain significant amounts of variance in a range of behaviors: naming of real words, lexical decision, and pseudoword reading. The measures from the toolkit either outperformed or were on par with the best available consistency measures, in terms of explaining variance in data from the English Lexicon Project. However, the strongest test of the validity of the toolkit also highlights one of its greatest contributions, which is its ability to account for behaviors in pseudoword tasks. Because a foundational assumption is that the sublexical system is used for generating spellings/readings in an online fashion, pseudoword reading and spelling tasks are arguably the most informative for revealing what knowledge is actually represented at the sublexical level – if people make use of a phoneme-grapheme mapping to read or spell a pseudoword, by definition that mapping is productive within the sublexical system. Here we successfully used the toolkit to account for the majority of the variance in the frequency of alternative pronunciations in a pseudoword reading task, helping explain why a pseudoword like DRICHE, for example, was more often pronounced /dɹiʃ/ than /dɹaɪʃ/ (Fig. 4; Table 4), with distinguishable contributions from different grain sizes, feedback (spelling consistency), and lexical contributions (in the form of phonological and orthographic neighbors). To the best of our knowledge, no systematic method has previously been published that allows for such analyses, largely because most available consistency measures are in the form of a list of real words with associated values – as such, they are not readily applicable to pseudowords, and therefore it has been difficult to quantitatively assess the sound–spelling mappings that are actually productive for literate English speakers.

The results presented in this manuscript provide examples of the broad range of potential applications of the toolkit for studying sublexical processing in written language. Moreover, the theoretical framework presented here, while grounded in decades of research on English spelling-sound consistency, is novel because it more deeply and systematically considers the implications of thinking of the sublexical system as an experience-dependent system. We conceive of sublexical knowledge as being acquired by experience with cross-modal mapping between phonemes and graphemes, and not as the internalization of rules or principles gleaned from formal instruction. Instead, it is an emergent network of connections between spoken and written forms organized in hierarchical fashion, from the whole-word level down to individual phonographemes (Fig. 1). As such, the toolkit is not intended to capture the *entirety* of what people know about reading and spelling, which also incorporates lexical, morphological, syntactic, and other dimensions of knowledge that here we define as being external to the sublexical system.

The details presented here about how the toolkit was developed, as well as practical guidance on how to use its functions, are likewise intended to illustrate the potential for addressing research questions that previously were more onerous or even intractable. Future research will continue to refine and expand the capabilities in a number of ways and will remain open access to researchers interested in developing alternative versions. There are many important, outstanding questions that can be addressed by applying the toolkit’s approach and/or by further enhancing the toolkit’s capabilities. A non-exhaustive list of possibilities includes: measuring consistency at other grain sizes (oncleus, biphone-bigrapheme units, etc.); incorporating other forms of knowledge such as morphology; developing accent-specific corpora (e.g., mapping standard spellings to particular regional pronunciations); probing the developmental trajectory of sound–spelling mappings (e.g., as new words enter the child’s lexicon, how does consistency change?); developing an orthographic counterpart to the phonological parsing Maximum Onset Principle; adjudicating between the importance of phonographeme frequency versus consistency (related to the issue of “feedforward” versus “feedback” processing); assessing errors in reading or spelling; probing the sublexical processing of special populations such as neuro-atypical individuals (e.g., in dyslexia or aphasia) or bilingual individuals; testing alternative position coding schema (e.g., serial position versus both-ends); comparing type- and token-weighted frequency measures; investigating sublexical representations through neuroimaging studies; etc. All of these are achievable either with the current version of the toolkit, by expanding the corpora to include more words, and/or by altering how the words are parsed (phonologically and/or orthographically). In essence, the hope is that this framework establishes a consistent and reliable platform to address these types of issues in a new, systematic way.

## Figures and Tables

**Fig. 6:**
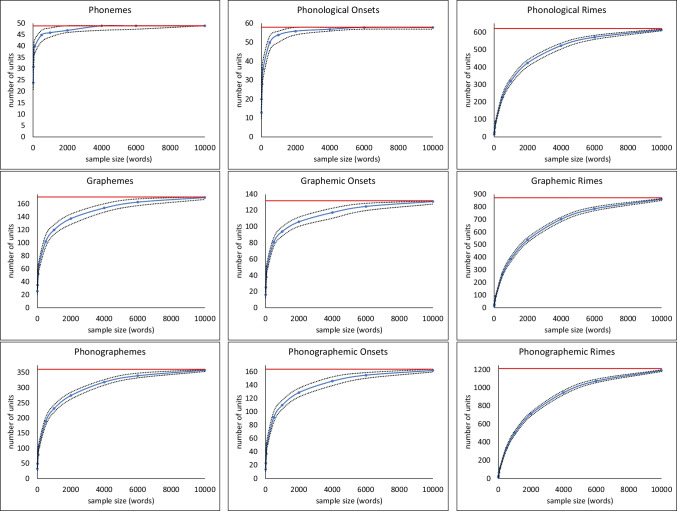
The increase in the number of sublexical units occurring at least twice (*y*-axis) as the corpus size increases (*x*-axis). Corpora were randomly resampled 100 times each with sizes of *n* = 10, 20, 50, 100, 500, 1000, 2000, 4000, 6000, and 10000 words. *Top row*: phonological units; *middle row*: graphemic units; *bottom row*: phonographemic units. *Left column*: phonographeme level; *middle and right columns*: onset and rime level, respectively. The *solid blue line* reflects the mean number of units across the 100 iterations per sample size; dashed lines reflect the 95% confidence interval. The *horizontal red line* reflects the number of units actually observed in the full corpus of 13338 words

**Fig. 7:**
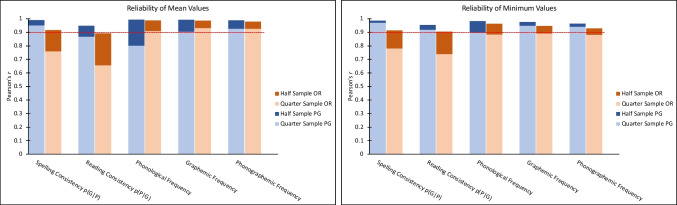
The height of the *light-colored* part of the bars indicates the estimated reliability when using only a quarter of the corpus (on average across 100 random iterations). The *dark-colored* part of the bars indicates the increase in reliability when using half of the corpus instead of only a quarter. *Blue*: PG (phonographeme) level; *orange*: OR (onset/rime) level. *Left panel*: correlations between mean values; *right panel*: correlations between minimum values. The *dashed red line* marks *r* = 0.9, indicating excellent internal validity

## Appendix 1

Appendix Table 6

**Table 6:** Table of phoneme-grapheme correspondences for vowels occurring at least 1% of the time and in at least two different word forms

Phoneme	Graphemes	Word Initial	Syllable Initial	Medial	Syllable Final	Word Final
æ	A	*a* * ba cus*	*pi * *a** no*	*c* *a**t*	*a c* *a** de my*	*y* *a*
	A_E	*A**nn* *e*	*Di * *a**n* *e*	*b* *a**ch* *e** lor*	*--*	*--*
	AU	*au* *nt*	*--*	*dr* *au**ght*	*l* *au** ghing*	*--*
	EAH	*--*	*--*	*--*	*--*	*y* *eah*
aɪ	AI	*--*	*--*	*--*	*ha w* *ai** i*	*ch* *ai*
	AI_E	*ai**sl* *e*	*--*	*--*	*--*	*--*
	AY	*ay* * a to llah*	*--*	*--*	*m* *ay** a*	*--*
	AYE	*aye*	*--*	*--*	*--*	*--*
	EI	*ei* * ther*	*--*	*--*	*n* *ei** ther*	*fah ren h* *ei**t*
	EYE	*eye*	*--*	*--*	*--*	*wa ll* *eye*
	I	*i*	*au thor * *i**zed*	*b* *i**nd*	*l* *i** bra ry*	*p* *i*
	I_E	*i**c* *e*	*me mor * *i**z* *e*	*pr* *i**z* *e*	*--*	*--*
	IA	*--*	*--*	*--*	*d* *ia** mond*	*--*
	IE	*--*	*--*	*a ll* *ie**s*	*h* *ie** ro glyph*	*p* *ie*
	IGH	*--*	*--*	*l* *igh**t*	*h* *igh** light*	*s* *igh*
	UY	*--*	*--*	*b* *uy**s*	*b* *uy** ing*	*g* *uy*
	Y	*--*	*--*	*--*	*c* *y** cle*	*b* *y*
	Y_E	*--*	*--*	*t* *y**p* *e*	*--*	*--*
	YE	*--*	*--*	*--*	*--*	*b* *ye*
aʊ	AU	*--*	*--*	*sau er kr* *au**t*	*s* *au** di*	--
	O	*o* * ur*	*--*	*--*	*sc* *o** ur*	--
	OU	*ou* *st*	*through * *ou**t*	*p* *ou**t*	*gr* *ou** chy*	*th * *ou*
	OU_E	*ou**nc* *e*	*--*	*bl* *ou**s* *e*	*--*	--
	OUGH	*--*	*--*	*dr* *ough**t*	*--*	*pl* *o**ugh*
	OW	*ow*	*--*	*cr* *ow**d*	*p* *ow** der*	*br* *ow*
ɑ	A	*a* *l mond*	*ko * *a** la*	*b* *a**rn*	*s* *a** ga*	*sp* *a*
	A_E	*--*	*en tour * *a**g* *e*	*l* *a**rg* *e*	*--*	--
	AH	*--*	*--*	*--*	*--*	*sh* *ah*
	AU	*au* *c tion*	*--*	*v* *au**lt*	*n* *au** ti cal*	--
	AW	*aw* * ning*	*--*	*--*	*str* *aw** berry*	*cole sl* *aw*
	E	*e* *n tre pre neur*	*--*	*r* *e**n dez vous*	*--*	--
	O	*o* * be lisk*	*bi * *o** lo gy*	*l* *o**ft*	*d* *o** llar*	--
	O_E	*o**m* *e** let*	*bri * *o**ch* *e*	*s* *o**lv* *e*	*--*	--
eɪ	A	*a* * ble*	*cre * *a** ted*	*b* *a**ss*	*bl* *a** tant*	--
	A_E	*a**c* *e*	*gra du * *a**t* *e*	*r* *a**t* *e*	*--*	--
	AE	*ae* * ge an*	*--*	*--*	*--*	*sun d* *ae*
	AI	*ai* *m*	*li * *ai** son*	*w* *ai**t*	*d* *ai** sy*	--
	AI_E	*ai**d* *e*	*--*	*co c* *ai**n* *e*	*--*	--
	AY	*ay* * cock*	*--*	*al w* *ay**s*	*m* *ay** be*	*tr* *ay*
	E	*--*	*car bur * *e** tor*	*--*	*d* *e** but*	*ca f* *e*
	EA	*--*	*--*	*st* *ea**k*	*br* *ea** king*	*y* *ea*
	EI	*--*	*--*	*f* *ei**gn*	*h* *ei** nous*	--
	EIGH	*eigh* *t*	*--*	*w* *eigh**t*	*n* *eigh** bor*	*sl* *eigh*
	ET	*--*	*--*	*--*	*--*	*ba ll* *et*
	EY	*--*	*--*	*--*	*pr* *ey** ing*	*th* *ey*
ə	A	*a* * gain*	*gi * *a**nt*	*hu m* *a**n*	*al ph* *a** bet*	ca ro li na
	A_E	*--*	*en cour * *a**g* *e*	*ma n* *a**g* *e*	*--*	--
	AH	*--*	*--*	*--*	*--*	chee tah
	E	*e* * ffect*	*cli * *e**nt*	*ham l* *e**t*	*con s* *e** quence*	gen re
	E_E	*--*	*sci * *e**nc* *e*	*pre s* *e**nc* *e*	*--*	--
	I	*--*	*de * *i** ty*	*a n* *i** mal*	*de f* *i** nite*	--
	I_E	*--*	*--*	*en g* *i**n* *e*	*--*	--
	IA		*ha wai * *ia**n*	*--*	*par l* *ia** ment*	fu schia
	O	*o* *b sess*	*bay * *o** net*	*fe l* *o**n*	*di n* *o** saur*	--
	OU	*--*	*joy * *ou**s*	*hei n* *ou**s*	*jea l* *ou** sly*	--
	U	*u* * pon*	*tri * *u**mph*	*au g* *u**st*	*i ll* *u** strate*	--
	non-linear E	*--*	*ax l* *e*	*hur dl* *e*	*--*	--
ɛ	A	*a* * ny*	*fe bru * *a** ry*	*sc* *a**red*	*em b* *a** rrass*	--
	A_E	*--*	*--*	*c* *a**r* *e*	*--*	--
	AE	*ae* * ri al*	*--*	*--*	*--*	--
	AI	*ai* *r*	*--*	*a g* *ai**n*	*pr* *ai** rie*	--
	E	*e* *c cen tric*	*no * *e**l*	*a ff* *e**ct*	*r* *e** bel*	--
	E_E	*e**dg* *e*	*cay * *e**nn* *e*	*d* *e**ns* *e*	*--*	--
	EA	*--*	*--*	*br* *ea**d*	*tr* *ea** sure*	--
	IE	*--*	*--*	*fr* *ie**nd*	*--*	--
ɚ	A	*--*	*cow * *a**rd*	*pi ll* *a**r*	*--*	--
	E	*e* *r go*	*flow * *e**r*	*po v* *e**r ty*	*--*	--
	E_E	*--*	*con ci * *e**rg* *e*	*v* *e**rg* *e*	*--*	--
	EA	*ea* *rl*	*--*	*y* *ea**rn*	*--*	--
	HE	*he* *rb*	*--*	*she ph* *e**rd*	*--*	--
	I	*i* *rk*	*e lix * *i**r*	*f* *i**rm*	*--*	--
	O	*o* *r i gi nal*	*may * *o**r*	*spon s* *o**r*	*--*	--
	OU	*--*	*--*	*a dj* *ou**rn*	*--*	--
	U	*u* *r ban*	*so * *u**r*	*b* *u**rn*	*--*	--
	U_E	*u**rg* *e*	*--*	*cul t* *u**r* *e*	*--*	--
	non-linear E	*--*	*py r* *e*	*o gr* *e*	*--*	--
i	E	*e* * mo tion*	*hyena*	*her cu l* *e**s*	*ro d* *e** o*	*re ci p* *e*
	E_E	*e**v* *e*	*--*	*sc* *e**n* *e*	*--*	--
	EA	*ea* *ch*	*--*	*wr* *ea**th*	*s* *ea** son*	*p* *ea*
	EA_E	*ea**s* *e*	*--*	*l* *ea**gu* *e*	*--*	*ii*
	EE	*ee* *l*	*--*	*f* *ee**t*	*st* *ee** ple*	*fl* *ee*
	EE_E	*--*	*--*	*sl* *ee**v* *e*	*--*	--
	EI	*ei* * ther*	*--*	*re c* *ei**pt*	*c* *ei** ling*	--
	EI_E	*--*	*--*	*de c* *ei**v* *e*	*--*	--
	EY	*--*	*--*	*--*	*vo ll* *ey** ball*	*tur k* *ey*
	I	*--*	*cour * *i** er*	*p* *i**z za*	*cham p* *i** on*	*chi l* *i*
	I_E	*--*	*na * *i**v* *e*	*e l* *i**t* *e*	*--*	*--*
	IE	*--*	*hurr * *ie**d*	*gr* *ie**f*	*me d* *ie** val*	*pix * *ie*
	IE_E	*--*	*--*	*re l* *ie**v* *e*	*--*	--
	Y	*--*	*worr * *y** ing*	*--*	*po n* *y** tail*	*ug l* *y*
ɪ	A_E	*--*	*cour * *a**g* *e*	*ba gg* *a**g* *e*	*--*	--
	E	*e* * ffi cient*	*--*	*ja ck* *e**t*	*a m* *e** thyst*	--
	EA	*ea* *r*	*--*	*cl* *ea**r*	*w* *ea** ry*	--
	EE	*--*	*--*	*d* *ee**r*	*ch* *ee** ry*	--
	HI	*--*	*ve * *hi** cle*	*--*	*--*	--
	I	*i* * cky*	*co * *i**n cide*	*f* *i**t*	*t* *i** ckle*	--
	I_E	*--*	*ur * *i**n* *e*	*g* *i**v* *e*	*--*	--
	Y	*--*	*--*	*g* *y**m*	*gl* *y** cer in*	--
ju	EU	*eu* * ca lyp tus*	*--*	*f* *eu**d*	*f* *eu** dal*	--
	EW	*--*	*--*	*--*	*--*	few
	EWE	*ewe*	*--*	*--*	*--*	--
	HU	*hu* * man*	*--*	*--*	*--*	--
	U	*u* * ni corn*	*sol * *u** ble*	*a cc* *u**sed*	*h* *u** mor*	*me n* *u*
	U_E	*u**s* *e*	*vol * *u**m* *e*	*m* *u**s* *e*	*--*	--
	UE	--	--	*c* *ue**s*	*--*	*ar g* *ue*
jʊ	EU	*eu* * rope*	*--*	*--*	*--*	--
	U	*u* * ra ni um*	*--*	*c* *u**red*	*c* *u** rate*	--
	U_E	--	*--*	*p* *u**r* *e*	*--*	--
oʊ	EAU	*--*	*--*	*--*	*--*	*bu r* *eau*
	O	*o* * bey*	*coy * *o** te*	*h* *o**ld*	*s* *o** cial*	*tri * *o*
	O_E	*o**l* *e*	*ca sser * *o**l* *e*	*cl* *o**n* *e*	*--*	--
	OA	*oa* *k*	*--*	*c* *oa**l*	*c* *oa** ster*	*co c* *oa*
	OE	--	--	*p* *oe**m*	*j* *oe** y*	*fl* *oe*
	OH	*oh*	*--*	*--*	*--*	--
	OU	--	--	*s* *ou**l*	*--*	*al th* *ough*
	OUGH	--	*thor * *ough** ly*	*--*	*d* *ough** nut*	*--*
	OW	*ow* *n*	*--*	*sh* *ow**n*	*b* *ow** ling*	*gr* *ow*
	OWE	*owe*	*--*	*--*	*--*	--
ɔ	A	*a* *l most*	*--*	*w* *a**rt*	*w* *a** rri or*	--
	AU	*au* * ra*	*--*	*di no s* *au**r*	*l* *au** rel*	--
	AUGH	*--*	*--*	*c* *augh**t*	*sl* *augh** ter*	--
	AW	*aw* * ful*	*--*	*dr* *aw**l*	*cr* *aw** ler*	*cl* *aw*
	AWE	*awe* * some*	*--*	*dr* *awe**r*	*--*	--
	O	*or* * phan*	*ex tra * *o**r di na ry*	*p* *o**rt*	*a d* *o** ra ble*	--
	O_E	*o**r* *e*	*--*	*ch* *o**r* *e*	*--*	--
	OA	*oa* *r*	*--*	*b* *oa**r*	*--*	--
	OO	*--*	*--*	*p* *oo**r*	*fl* *oo** ring*	--
	OU	*--*	*--*	*y* *ou**r*	*p* *ou** ring*	--
	OUGH	*ough* *t*	*--*	*b* *ough**t*	*--*	--
ɔɪ	OI	*oi* *nk*	*--*	*p* *oi**nt*	*se qu* *oi** a*	*k* *oi*
	OI_E	--	--	*n* *oi**s* *e*	--	--
	OY	*oy* * ster*	*--*	*b* *oy**s*	*l* *oy** al*	*t* *oy*
	OY_E	--	*--*	*gar g* *oy**l* *e*	*--*	--
u	EU	*--*	*--*	*sl* *eu**th*	*ma n* *eu** ver*	*--*
	EW	*--*	*--*	*l* *ew**d*	*s* *ew** er*	*br* *ew*
	O	*--*	*--*	*t* *o**mb*	*m* *o** vie*	*d* *o*
	O_E	*--*	*--*	*pr* *o**v* *e*	*--*	*--*
	OE	*--*	*--*	*sh* *oe**s*	*sh* *oe** lace*	*ca n* *oe*
	OO	*oo* * dles*	*--*	*b* *oo**th*	*d* *oo** dle*	*z* *oo*
	OO_E	*oo**z* *e*	*--*	*m* *oo**se*	*--*	--
	OOH	*ooh*	*--*	--	*--*	*p* *ooh*
	OU	*--*	*--*	*s* *ou**p*	*r* *ou** tine*	*y* *ou*
	OU_E	*--*	*--*	*r* *ou**g* *e*	--	*--*
	U	*--*	*--*	*tr* *u**th*	*c* *u** ckoo*	*fl* *u*
	U_E	*--*	*--*	*spr* *u**c* *e*	--	*--*
	UE	*--*	*--*	*t* *ue**s day*	*bl* *ue** be rry*	*gl* *ue*
	UI	*--*	*--*	*fr* *ui**t*	*br* *ui** ses*	*--*
	UI_E	*--*	*--*	*cr* *ui**s* *e*	--	*--*
ʌ	O	*o* *n ion*	--	*fr* *o**m*	*a n* *o** ther*	--
	O_E	*--*	*--*	*s* *o**m* *e*	--	--
	OU	*--*	*--*	*t* *ou**ch*	*c* *ou** sin*	--
	U	*u* * gly*	*blow * *u**p*	*g* *u**m*	*j* *u** ggle*	--
	U_E	--	--	*gr* *u**dg* *e*	--	*--*
	UH	*uh*	--	--	--	*d* *uh*
	non-linear E	--	--	*a ny on* *e*	--	*--*

## Appendix 2

Appendix Table 7

**Table 7:** Table of phoneme-grapheme correspondences for consonants occurring at least 1% of the time and in at least two different word forms

Phoneme	Graphemes	Word Initial	Syllable Initial	Medial	Syllable Final	Word Final
b	B	*b* *ag*	*a * *b**le*	*jo* *b**s*	*lo* *b** ster*	*we* *b*
	BB	*--*	*lo * *bb**y*	*ro* *bb**ed*	*--*	*e* *bb*
d	D	*d* *og*	*ban * *d**age*	*a* *d**ze*	*a* *d** mire*	*re* *d*
	DD	*--*	*che * *dd**ar*	*o* *dd**s*	*to* *dd** ler*	*a* *dd*
	ED	*--*	*--*	*hun dr* *ed**s*	*ti r* *ed** ness*	*train* *ed*
ð	TH	*th* *us*	*lea * *th**er*	*clo* *th**es*	*smoo* *th** ness*	*ba* *th**e*
dʒ	D	*--*	*ar * *d**u ous*	*--*	*--*	*--*
	DG	*--*	*ba * *dg**er*	*--*	*ju* *dg**e ment*	*bu* *dg**e*
	DI	*--*	*sol * *di**er*	*--*	*--*	*--*
	DJ	*--*	*a * *dj**ust*	*--*	*--*	*--*
	G	*g* *in ger*	*stran * *g**er*	*chan* *g**ed*	*ve* *g**e ta ble*	*a* *g**e*
	GG	*--*	*su * *gg**est*	*--*	*--*	*--*
	GI	*--*	*re * *gi**on*	*--*	*--*	*--*
	J	*j* *ump*	*con * *j**ure*	*--*	*--*	*--*
f	F	*f* *ast*	*so * *f**a*	*cle* *f**t*	*a* *f** ter*	*chie* *f*
	FF	*--*	*o * *ff**er*	*cu* *ff**s*	*di* *ff**e rence*	*cli* *ff*
	GH	*--*	*tou * *gh**er*	*drau* *gh**t*	*lau* *gh** ter*	*rou* *gh*
	LF	*--*	*--*	*--*	*--*	*ha* *lf*
	PH	*ph* *an tom*	*tro * *ph**y*	*s* *ph**ere*	*o* *ph** thal mo lo gy*	*gra* *ph*
g	G	*g* *o*	*a * *g**ain*	*le* *g**s*	*co* *g** ni tion*	*ra* *g*
	GG	*--*	*nu * *gg**et*	*be* *gg**ed*	*e* *gg** nog*	*e* *gg*
	GH	*gh* *oul*	*spa * *gh**e tti*	*--*	*--*	*ar* *gh*
	GU	*gu* *ard*	*be * *gu**ile*	*ro* *gu**es*	*--*	*lea* *gu**e*
h	H	*h* *ave*	*in * *h**e rit*	*--*	*--*	*--*
	WH	*wh* *ole*	*--*	*--*	*--*	*--*
j	I	*--*	*brill * *i**ant*	*be ha v* *i**or*	*--*	*--*
	J	*--*	*ha lle lu * *j**ah*	*f* *j**ord*	*--*	*--*
	LL	*--*	*tor ti * *ll**a*	*--*	*--*	*--*
	Y	*y* *outh*	*be * *y**ond*	*--*	*--*	*--*
k	C	*c* *at*	*se * *c**ond*	*a* *c**t*	*ar* *c** tic*	*dis* *c*
	CC	*--*	*o * *cc**ur*	*--*	*--*	*--*
	CH	*ch* *oir*	*an * *ch**or*	*s* *ch**ool*	*te* *ch** nique*	*mo nar* *ch*
	CK	*--*	*bra * *ck**et*	*ba* *ck**s*	*ni* *ck** name*	*lu* *ck*
	K	*k* *ale*	*snor * *k**el*	*ban* *k**s*	*than* *k** ful*	*loo* *k*
	LK	*--*	*ta * *lk**ing*	*fo* *lk**s*	*--*	*yo* *lk*
	Q	*q* *uack*	*fre * *q**uent*	*s* *q**ueak*	*--*	*--*
	QU	*qu* *iche*	*li * *qu**or*	*mo s* *qu**i to*	*--*	*pla* *qu**e*
	X	*--*	*--*	*--*	*e* *x** cite*	*--*
ks	X	*--*	*--*	*mi* *x**ed*	*e* *x** change*	*he* *x*
kʃ	X	*--*	*--*	*--*	*se* *x** ual*	*--*
	XI	*--*	*--*	*--*	*ob no* *xi** ous*	*--*
l	L	*l* *ab*	*at * *l**as*	*se* *l**f*	*a* *l** bum*	*pa* *l*
	LL	*--*	*ba * *ll**oon*	*do* *ll**s*	*ha* *ll** way*	*fi* *ll*
m	LM	*--*	*sa * *lm**on*	*psa* *lm**s*	*--*	*pa* *lm*
	M	*m* *ouse*	*le * *m**on*	*s* *m**ug*	*a* *m** ber*	*stor* *m*
	MB	*--*	*plu * *mb**ing*	*nu* *mb**s*	*thu* *mb** tack*	*co* *mb*
	MM	*--*	*su * *mm**er*	*hu* *mm**ed*	*--*	*u* *mm*
	MN	*--*	*--*	*da* *mn**ed*	*--*	*hy* *mn*
n	GN	*gn* *at*	*si * *gn**age*	*rei* *gn**ed*	*a ssi* *gn** ment*	*be ni* *gn*
	KN	*kn* *ack*	*un * *kn**own*	*--*	*--*	*--*
	MN	*--*	*--*	*--*	*--*	*--*
	N	*n* *ail*	*va * *n**ish*	*wa* *n**t*	*fa* *n** cy*	*ma* *n*
	NN	*--*	*pe * *nn**y*	*ca* *nn**ed*	*pe* *nn** syl van ia*	*i* *nn*
	PN	*pn* *eu mon ia*	*--*	*--*	*--*	*--*
am	GN	*--*	*la sa * *gn**a*	*--*	*--*	*--*
	N	*--*	*se * *n**or*	*--*	*--*	*--*
ŋ	N	*--*	*--*	*ba* *n**k*	*a* *n** kle*	*--*
	NG	*--*	*--*	*a* *ng**st*	*ga* *ng** ster*	*ki* *ng*
p	P	*p* *ace*	*u * *p**on*	*s* *p**y*	*tem* *p** ta tion*	*cu* *p*
	PP	*--*	*ha * *pp**y*	*ri* *pp**ed*	*--*	*schle* *pp*
r	R	*r* *ight*	*theo * *r**y*	*c* *r**ow*	*to* *r** na do*	*ai* *r*
	RH	*rh* *yme*	*--*	*--*	*--*	*--*
	RR	*--*	*so * *rr**y*	*o ccu* *rr**ed*	*cu* *rr** ent*	*pu* *rr*
	RRH	*--*	*ci * *rrh**o sis*	*--*	*he mo* *rrh** age*	*my* *rrh*
	WR	*wr* *ite*	*un * *wr**ap*	*han d* *wr**i ting*	*--*	*--*
s	C	*c* *ent*	*fan * *c**y*	*fa* *c**ed*	*i* *c**e berg*	*dan* *c**e*
	ES	*--*	*--*	*--*	*--*	*flak* *es*
	S	*s* *ent*	*fru * *s**trate*	*wai* *s**t*	*mi* *s** chief*	*ga* *s*
	SC	*sc* *ent*	*cre * *sc**ent*	*--*	*--*	*re mi ni* *sc**e*
	SS	*--*	*go * *ss**ip*	*ki* *ss**ed*	*cla* *ss** room*	*cro* *ss*
	ST	*--*	*whi * *st**le*	*--*	*--*	*--*
	Z	*--*	*pret * *z**el*	*blit* *z**ed*	*mit* *z** vah*	*dit* *z*
ʃ	C	*--*	*o * *c**ean*	*--*	*--*	*li cor i* *c**e*
	CH	*ch* *ef*	*ma * *ch**ine*	*--*	*--*	*ni* *ch**e*
	CI	*--*	*an * *ci**ent*	*--*	*--*	*--*
	S	*s* *u gar*	*en * *s**ure*	*--*	*--*	*--*
	SCH	*sch* *muck*	*e * *sch**ew*	*--*	*--*	*kir* *sch*
	SI	*--*	*man * *si**on*	*--*	*--*	*--*
	SH	*sh* *ake*	*mar * *sh**al*	*pu* *sh**ed*	*marsh mellow*	*wi* *sh*
	SS	*--*	*ti * *ss**ue*	*--*	*--*	*--*
	SSI	*--*	*mi * *ssi**on*	*--*	*--*	*--*
	T	*--*	*i ni * *t**i ate*	*--*	*--*	*--*
	TI	*--*	*mar * *ti**al*	*--*	*--*	*--*
t	ED	*--*	*--*	*--*	*--*	*dress* *ed*
	T	*t* *oad*	*men * *t**al*	*ra* *t**s*	*sel* *t** zer*	*ha* *t*
	TT	*--*	*mi * *tt**en*	*pu* *tt**s*	*--*	*mu* *tt*
tʃ	CH	*ch* *ain*	*or * *ch**ard*	*rea* *ch**ed*	*hen* *ch** man*	*ea* *ch*
	T	*--*	*pic * *t**ure*	*--*	*--*	*--*
	TCH	*--*	*ke * *tch**up*	*i* *tch**ed*	*wa* *tch** ful*	*wre* *tch*
	TI	*--*	*ques * *ti**on*	*--*	*--*	*--*
v	V	*v* *ine*	*na * *v**y*	*lo* *v**es*	*so* *v**e reign*	*sa* *v**e*
	LV	*--*	*--*	*ca* *lv**es*	*--*	*ha* *lv**e*
w	O	*o* *ne*	*a ny * *o**ne*	*n* *o**ir*	*--*	*--*
	U	*--*	*si lho * *u**ette*	*pen g* *u**in*	*--*	*--*
	W	*w* *eed*	*al * *w**ays*	*s* *w**itch*	*--*	*--*
	WH	*wh* *ale*	*no * *wh**ere*	*--*	*--*	*--*
z	ES	*--*	*--*	*--*	*wedn* *es** day*	*mov* *es*
	S	*--*	*wea * *s**el*	*clo* *s**ed*	*cha ri* *s** ma*	*wa* *s*
	SS	*--*	*de * *ss**ert*	*--*	*--*	*--*
	X	*x* *y lo phone*	*an * *x**i e ty*	*--*	*--*	*--*
	Z	*z* *e bra*	*wi * *z**ard*	*gla* *z**ed*	*--*	*ma* *z**e*
	ZZ	*--*	*bli * *zz**ard*	*bu* *zz**ed*	*gri* *zz** ly*	*ja* *zz*
ʒ	G	*--*	*ba rra * *g**es*	*--*	*--*	*rou* *g**e*
	S	*--*	*plea * *s**ure*	*--*	*--*	*--*
	SI	*--*	*a * *si**a*	*--*	*--*	*--*
	Z	*--*	*sei * *z**ure*	*--*	*--*	*--*
θ	TH	*th* *ank*	*au * *th**or*	*my* *th**s*	*a ri* *th** me tic*	*wrea* *th*

## Appendix 3: Evaluation of corpus size

Two sets of analyses were conducted to assess the adequacy of the corpus size in the latest version of the English Sublexical Toolkit (version 1.1), which contains 13,388 words.

## Methods

### Analysis C1: Relationship between corpus size and number of sublexical units

First, we sought to estimate how many sublexical units (phonemes, graphemes, and phonographemes) are encountered as the corpus size increases. To do this, we used resampling methods – specifically, the full Sublexical Toolkit version 1.1 corpus of 13,338 words was repeatedly resampled across sample sizes ranging from *n* = 10 words up to* n* = 10,000 words. The number of units appearing in each subsample was counted, and this process was repeated for 100 iterations (i.e., 100 randomly drawn subsamples of words for each sample size). Only units that occur at least twice in the full corpus were counted, and the number of these is also presented as a “benchmark” for comparing the different corpus sizes (i.e., the results are presented as the *percentage* of the units in the full 13,338 word corpus, excluding units that may be specific to a single lexical item).

### Analysis C2: Reliability of the Sublexical Toolkit measures

Second, we also sought to determine the reliability of each of the five toolkit measures (Spelling Consistency p(G|P), Reading Consistency p(P|G), Phoneme Frequency, Grapheme Frequency, and Phonographeme Frequency). We were interested in two statistics: given tables of probabilities/frequencies informed by subsets of the full corpus, what are (a) the proportion of words *not used to inform the tables* that can be successfully mapped (i.e., all segments parsed, and consistency/frequency values assigned), and (b) the degree to which the mean and minimum values for successfully mapped words are reliable. We used a cross-validation procedure to obtain these measures, as detailed below. This procedure also serves to highlight the functions of the R-based Sublexical Toolkit (see materials on OSF for details on how to use each of the functions).

Details of the procedure:

- Step One: The functions *map_PG* and *map_OR* take as input a list of words (spellings and pronunciations) and return as output matrices with the words parsed into phonemes and corresponding graphemes, at the phonographeme level (function *map_PG*) and the onset/rime level (function *map_OR*). These functions were applied to the full 13,338 word corpus of the Sublexical Toolkit version 1.1, and the outputs were randomly sub-sampled to create corpora one half or one quarter the full size (6669 words or 3335 words). This process was repeated for 100 iterations, generating 200 *pairs* of corpora based on independent split-halves of the full corpus (100 at the phonographeme level and 100 at the onset/rime level), and 200 *pairs* of corpora based on independent split-quarters of the full corpus.
- Step Two: The function *make_tables* takes as input words already parsed into phonemes and corresponding graphemes (i.e., the output of the functions *map_PG* or *map_OR*) and returns as output tables of probabilities and frequencies. This function was applied to each of the sub-sampled corpora produced in Step One, producing pairs of tables with independent estimates of probabilities and frequencies (e.g., one table based on words one through 3335 and a second table based on words 3336 through 6670). In other words, we obtained pairs of tables that differ in the probability and frequency values because they were informed by independent corpora.
- Step Three: The function *map_values* takes as input a list of words (phonemes and graphemes) plus a table of probabilities and frequencies (i.e., the output of function *make_tables*), and returns as output matrices of parsed words with values corresponding to the sublexical mappings (e.g., the Spelling Consistency p(G|P) for each mapping in the word). Each of the tables output in Step Two were given in turn as input to *map_values*, effectively providing multiple independent estimates of the consistency and frequency values for all 13,338 words in the full corpus. Some words could not be scored (e.g., a table based on a corpus lacking the word “yacht” fails to then score the word “yacht” because it has no entry for the grapheme ACH) – the proportion of words that *were not* used to inform the table but could still be scored represents an estimate of how well the sub-sampled corpora generalizes to the broader lexicon.
- Step Four: The function *summarize_words* takes as input matrices in which words have been parsed and values applied (i.e., the output of *map_values*) and provides as output descriptive statistics for the parameter(s) of interest. This function was applied to each of the outputs from Step Three, to extract the mean and minimum values for all five Sublexical Toolkit measures and all words in the full corpus. Finally, the Pearson correlation between each matched pair of measures was computed to estimate reliability. Note that the final result of this procedure was, for each statistic of interest, 100 estimates per level (phonographeme or onset/rime) and per corpus size (6669 words or 3335 words).

## Results

### Results C1: Relationship between corpus size and number of sublexical units

The results of the resampling analyses are presented below in Fig. 6. In each panel of the figure, the red line represents the number of units empirically observed in the full sample of 13,338 words. It can be seen that, overall, there are fewer phonological units than graphemic ones (compare top and middle rows), and more phonographemic units (i.e., mappings between phonemes and graphemes) than anything else (bottom row). Similarly, there are more onsets than singular phonemes (because the onsets may be clusters), and still more rimes (because of the many combinations of vowels with codas). In all cases, there is clearly an initial explosion of the number of units from the smallest corpus size (*n* = 10 words) up to around *n* = 500, and then a deceleration in the occurrence of new units as the corpus increases further. The lowest number of units is for phonemes – in fact, there are only 49 in the full corpus^9^, and typically all of them are encountered once the corpus size reaches *n* = 4000 words (Fig. 6 top left). There are slightly more phonological onsets, but it can be seen that all of these are reliably encountered once the corpus reaches *n* = 6000 words (Fig. 6 top center). Accordingly, it is the rime units that least approach an asymptote as the sample size increases (Fig. 6 right column); nonetheless, the vast majority of rime units are encountered by the time the corpus reaches *n* = 6000 words, and exceedingly few new rimes are encountered when increasing the sample size from 10000 to 13338 words (this can be seen by the very small ‘gap’ between the horizontal red line, depicting the number of units in the full corpus, and the final endpoint on the blue line for subsamples of 10000 words).

### Analysis C2: Reliability of the Sublexical Toolkit measures

The first set of results from this analysis is presented in Table 8, reporting the percentage of words in the full corpus (*n* = 13,338) that were successfully parsed and mapped when using tables of probabilities/frequencies informed by only half (6669 words) or a quarter (3335) of the corpus. From this it can be seen that half of the corpus is sufficient to parse 95.46% of the words that were *not* used to inform the tables, at the phonographeme level. This compares to 84.59% at the onset/rime level. This difference is of course due to the fact that a corpus may encounter the *constituents* of an onset or rime unit, without actually encountering the specific combination of those units that generate that rime. For example, a corpus made solely of the words CAT and CUP is sufficient to generate mappings at the sublexical level for the word CAP (because the medial A and final P have been encountered in the corpus), but *not* for the rime -AP (because that *combination* of vowel and coda is not present in either CAT or CUP). Regarding corpora only a quarter of the size, a somewhat lower percentage of words are successfully mapped at the phonographeme level (≈ 92% versus ≈ 95%), whereas the results for the onset/rime level decline more precipitously (≈ 75% vs. ≈ 85%). As a point of comparison, version 1.0 of the toolkit, based on *n* = 10650 words, successfully parses 99.00% of the words added for version 1.1 (i.e., 99.00% of the 2688 words on which it was *not* based).

**Table 8:** The percentage of words that were successfully parsed when informing the tables of probabilities/frequencies with only half or a quarter of the corpus (*excludes* the words used to inform the tables)

Level:	Half corpus		Quarter corpus	
	(*n* = 6669)	95% CI	(*n* = 3335)	95% CI
Phonographeme	95.46%	[94.12, 96.61]	91.92%	[90.52, 93.02]
Onset/Rime	84.59%	[91.48, 93.00]	74.70%	[73.09, 76.57]

The second set of results is depicted in Table 9 and Fig. 7 below. These indicate that split-half reliability (Fig. 7, dark blue for mean values and dark orange for minimum values) is excellent, above a Pearson’s *r* = 0.9 for all but one measure (*r* = 0.892 for reading consistency at the onset/rime level). As a further point of validation, reliability when using only a quarter of the corpus (Fig. 7, light blue for mean values and light orange for minimum values) is in the range of *r* = 0.66 (for reading consistency at the onset/rime level) to 0.95 (for spelling consistency at the phonographeme level). These results strongly suggest that the corpus of the Sublexical Toolkit (both versions 1.0 and 1.1) is sufficient to provide strong internal validity, for both the mean and minimum values of all five primary measures (spelling consistency, reading consistency, and frequencies of phonemes, graphemes, and phonographemes).

**Table 9:** The split-half reliability of the Sublexical Toolkit measures based on 100 iterations, measured with Pearson’s *r* correlations

	Phonographeme level				Onset & rime level			
Measure:	Mean	*95% CI*	Min	*95% CI*	Mean	*95% CI*	Min	*95% CI*
Spelling Consistency p(G|P)	0.989	*[0.984, 0.992]*	0.986	*[0.981, 0.989]*	0.917	*[0.898, 0.929]*	0.913	*[0.894, 0.922]*
Reading Consistency p(P|G)	0.948	*[0.937, 0.957]*	0.955	*[0.946, 0.963]*	0.892	*[0.876, 0.903]*	0.906	*[0.883, 0.915]*
Phoneme Frequency	0.993	*[0.995, 0.991]*	0.983	*[0.976, 0.988]*	0.987	*[0.986, 0.989]*	0.963	*[0.957, 0.967]*
Grapheme Frequency	0.992	*[0.991, 0.993]*	0.977	*[0.973, 0.981]*	0.986	*[0.984, 0.987]*	0.947	*[0.941, 0.952]*
Phonographeme Frequency	0.989	*[0.987, 0.990]*	0.964	*[0.960, 0.968]*	0.979	*[0.987, 0.990]*	0.928	*[0.922, 0.934]*

## Footnotes

1 See section “Empirical Validations” for new evidence regarding this question.

2 That is to say, the vowel /ɑ/ must map onto both O and AUGH for speakers with this particular merge, whereas those who maintain the distinction may map /ɑ/ to AUGH and /ɔ/ to O.

3 N.B.: the results of the analyses in this section reflect version 1.0 of the toolkit, whereas the internal validations presented in Appendix 3 draw upon comparisons between version 1.0 and version 1.1 of the toolkit (e.g., to assess the stability of the measures as the corpus size increases).

4 The discrepancy between the number of items coded for onset and for rime is due to 148 words with an empty onset position (i.e., all syllables are vowel-initial). It should be noted that for such words, the Sublexical Toolkit only provides Rime values. While Chee et al. (2020) do compute onset values for vowel-initial syllables, it is unclear what those reflect, especially given the fact they all have one fixed value regardless of the underlying phonemes or graphemes (e.g., the onset consistency for the EA in EAT is identical to that of EA in EARTH and the EE in EEL).

5 The step one model, with only surface and lexical variables, had a VIF ≈ 10.5 for OLD.

6 As a further verification of the Toolkit’s Phoneme_LgFreq measure, we also assessed the correlation between Naming RT and Accuracy and the phonotactic measures of Vaden et al. (2009). Those phonotactic measures showed the same pattern of association as the Toolkit’s: slower RT but higher accuracy for words with higher compared to lower phonotactic probability.

7 That is because the F/E measures (Plaut et al., 1996) by definition provide only one value per word, and the composite measures provided by Chee et al. (2020) are means.

8 The only significant negative associations were with the number of syllables and phoneme frequency. While the latter showed a slight negative association, it should be noted that its sign flipped from positive to negative in simultaneous regression, indicating that the relationship changes once controlling for the other variables.
